# Advances in Se Wide‐Bandgap Photovoltaics and Te_x_Se_1‐x_ SWIR Photodiodes

**DOI:** 10.1002/advs.77340

**Published:** 2026-09-03

**Authors:** Adam D. Alfieri, Deep Jariwala

**Affiliations:** ^1^ Electrical and Systems Engineering University of Pennsylvania Philadelphia Pennsylvania USA; ^2^ Institut Photovoltaïque d’Île de France (UMR 9006 IPVF) CNRS Palaiseau France

**Keywords:** indoor Photovoltaics, photovoltaics, selenium, SWIR photodiodes, tellurium

## Abstract

Elemental selenium (Se), tellurium (Te), and their alloys (Te_x_Se_1‐x_) have re‐emerged over the past decade as a promising materials system for low‐cost and sustainable thin‐film optoelectronic devices. In particular, Se has become a promising material for wide bandgap photovoltaics (PVs), and Te_x_Se_1‐x_ has arisen as a high‐performance thin‐film option for infrared (IR) photodetectors. In this review, we focus on recent developments in Se PVs and Te_x_Se_1‐x_ short‐wave IR (SWIR) photodiodes (PDs). We discuss the motivations for pursuing these materials, the fundamental properties, considerations for toxicity and sustainability, and the advances in materials science and device engineering. Finally, we discuss open challenges and underexplored opportunities for further development.

## Graphical abstract

In this review, fundamental properties and current challenges in Se photovoltaics (PVs) for tandem and indoor PVs and Te_x_Se_1‐x_ alloys for short wave infrared photodiodes are discussed. A case is made for the practicality of elemental chalcogenide optoelectronics, and a pathway toward resolving the key challenges in these respective technologies is outlined.

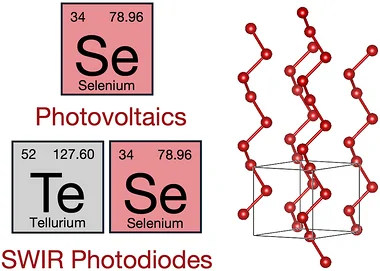

## 1 Introduction

Silicon (Si) has dominated the semiconductor industry for the better part of a century, so it is perhaps unexpected that the first great contributions to semiconductor physics were made with Se. In 1873, Willoughby Smith discovered photoconductivity in Se [1]. Ten years later, Charles Fritts made the first solid‐state PV from Se [2]. These discoveries helped lay the groundwork for the field of optoelectronics as we have come to know it. With the emergence of germanium (Ge) and Si in the mid‐20^th^ century, Se began to fall by the wayside. Research into Se and its alloys with Te for optoelectronics experienced a slight resurgence in the 1980s [3, 4, 5]. With the exceptions of applications in xerography and then X‐Ray detection [6, 7, 8, 9], this was not sustained, and interest in Se optoelectronics again faded.

In the past decade, over a century after Fritts made the first Se PV, Se and Te optoelectronics have re‐entered research circles. Wide bandgap PV materials have gained traction in recent years for applications in tandem and indoor PVs [10, 11, 12]. Se offers a combination of stability, simplicity, and steadily improving performance that makes it a promising long‐term alternative for wide bandgap PVs compared to toxic and often unstable lead halide perovskites (LHPs) [13, 14, 15, 16]. Meanwhile, growing demand for SWIR PDs motivates the development of materials with lower cost than epitaxial III‐V materials and lower toxicity than current alternatives: lead sulfide (PbS) and mercury telluride (HgTe) colloidal quantum dots (CQDs) [17]. Te_x_Se_1‐x_ fits these requirements, and initial works have shown the potential for high performance and spectral tunability [18, 19, 20, 21, 22, 23, 24]. We believe it is worth noting that, unlike previous periods of research on Se and Te, these materials are no longer in direct competition with crystalline Si (c‐Si), which has a bandgap too narrow for IPV applications and too wide for SWIR photodetection. The demand for new applications and lack of competition with c‐Si offer a more promising outlook for Se PVs and Te_x_Se_1‐x_ SWIR PDs.

Zhu et al. provided a nice review on Se PVs in 2019 [25], but a host of new results in the past several years and an increase in activity in the field merit an updated review. Additionally, the emergence of Te_x_Se_1‐x_ SWIR photodiodes (PDs) in 2023 and the research that has followed introduce another technologically important application of this materials system. Se and Te_x_Se_1‐x_ alloys share the same crystal structure, similar processing and materials chemistry, and similar optoelectronic properties and challenges. The focus on pure Se for PVs and alloys for SWIR PDs instead of pure Te reflects the trends in the respective research fields and the compositions with the best performance to date. Although materials science discoveries and device engineering advances do not always translate perfectly between Se PVs and Te‐rich Te_x_Se_1‐x_ PDs, we believe that improved overlap of these fields and enhanced understanding of the whole material system will be beneficial to the development of each type of device. We therefore treat these materials/devices in parallel and highlight the key similarities and differences. Here, we revisit basic optoelectronic properties and consider recent advances after a decade of renewed research in this system, and we argue that the promising performance, cost, stability, and low toxicity make this unique elemental system promising for thin film wide bandgap PVs (in tandem solar cells and indoor PVs) and SWIR PDs. During the course of submission and peer review we became aware of recent review papers on partially overlapping topics [21, 26, 27]. Our review differs from and complements these works in several ways, including but not limited to: (i) we treat the two fields together, identifying where advances in one field could translate to the others, (ii) we discuss in more depth the anisotropy on the crystal structure and its impact on not only device performance but also characterization, and caution against other current practices that may lead to inaccurate estimates of carrier lifetimes or mobilities, (iii) we provide a thorough and necessary discussion related to sustainability and toxicity, and (iv) we offer our opinions regarding necessary directions related to avoiding two‐step processing of Se given challenges related to metastable phases, which we view as an overlooked issue that could be associated with the open circuit voltage (*V_oc_*) loss.

**FIGURE 1:**
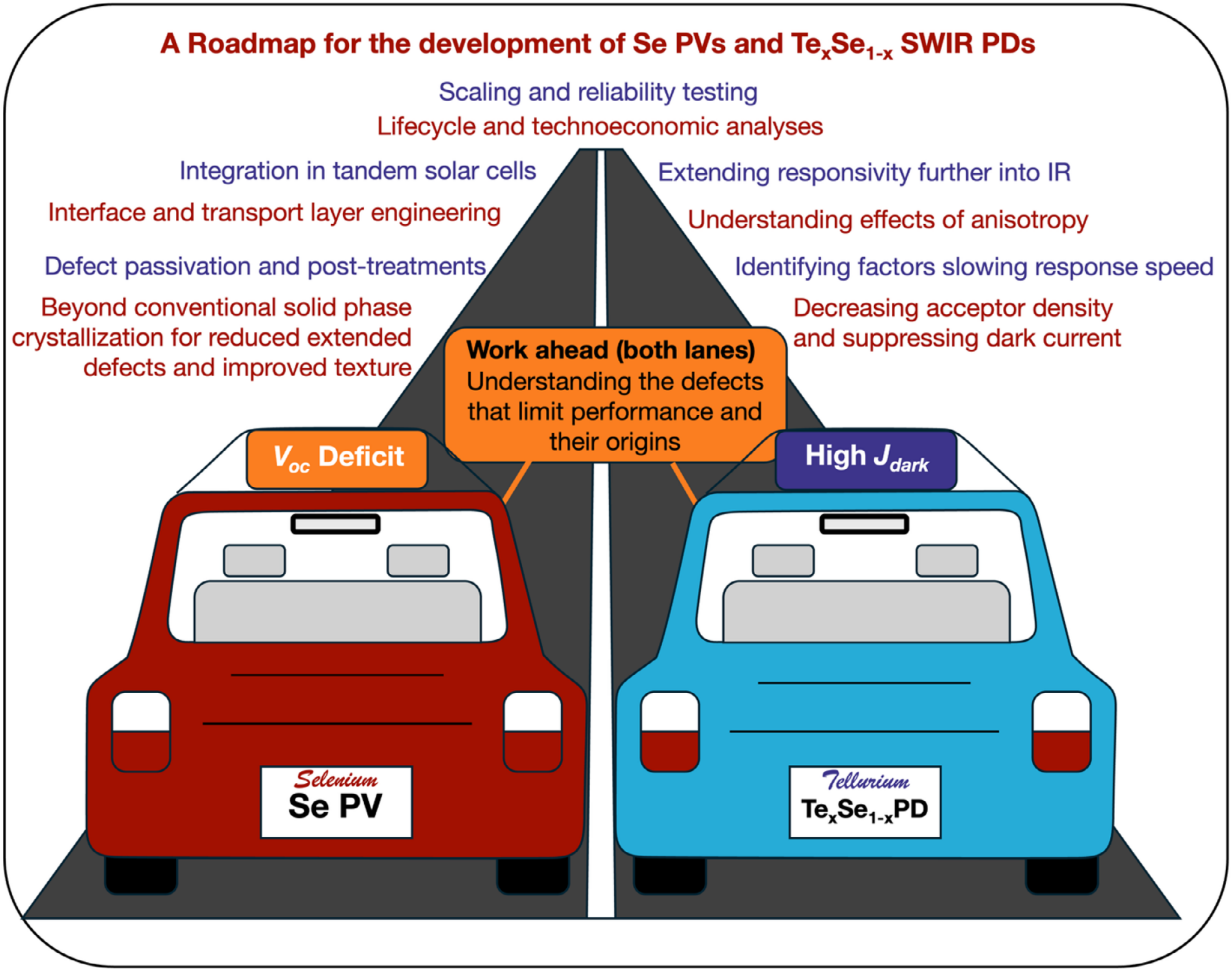
Visual roadmap for Se PVs and Te_x_Se_1‐x_ SWIR PDs. Se PVs are “weighed down” by a large open circuit voltage (*V_oc_*) deficit, while Te_x_Se_1‐x_ PDs suffer from a high dark current density (*J_dark_*). Initial work for both technologies will be to identify the defects responsible for the key performance losses and connect the defects to their materials processing (or potentially, intrinsic) origins. We list recommended paths for development of Se PVs and Te_x_Se_1‐x_ PDs on the left and right, respectively. Eventually, both technologies will require thorough lifecycle and technoeconomic analyses prior to work aimed at scaling for commercialization.

This review is structured as follows. First, we consider fundamental structural and optoelectronic properties, particularly the roles of polymorphs, anisotropy, and defects. We then detail the applications for which these materials are being considered in this review, along with practical considerations such as toxicity and material availability. Next, we focus on materials processing and device engineering before benchmarking Se solar and indoor PVs and Te_x_Se_1‐x_ SWIR PDs against other technologies of varying maturity levels. The choice to discuss applications before materials processing is unconventional but reflects our belief that materials processing must be considered with specific applications in mind. Finally, we offer an outlook, challenges, and conclusions section in which we offer our opinion on the current state of these fields, how the fields overlap, and what we view as the primary challenges. The visual roadmap in Figure 1 summarizes the outlook and conclusions of this work.

## 2 Fundamental Properties

### 2.1 Structure and Polymorphs

**FIGURE 2:**
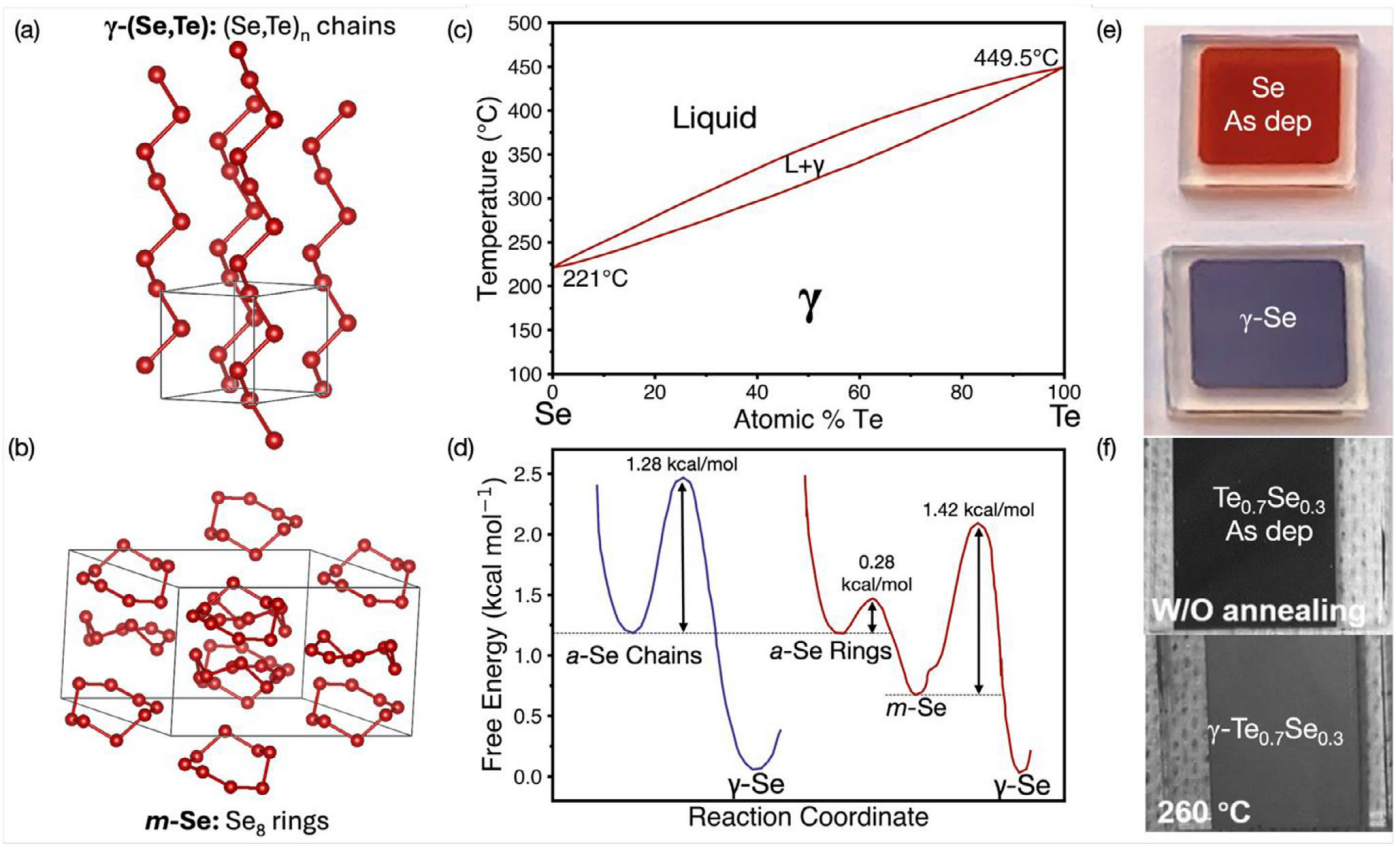
Structure of Se and Te_x_Se_1‐x_. (a) Crystal structure of γ‐Se, representative of γ‐Te_x_Se_1‐x_. (b) Crystal structure of metastable *m*‐Se. (c) Se‐Te phase diagram showing the complete solid solubility and low melting points of the system. Data reproduced from ref. [28]. (d) Differential scanning calorimetry measurements showing the crystallization paths of pure Se from chain‐like amorphous Se and ring‐like amorphous Se. Data reproduced from ref. [30]. (e) Pictures of vapor‐deposited Se films on glass substrates before (top) and after (bottom) crystallization. (f) Pictures of vapor‐deposited Te_0.7_Se_0.3_ films on glass substrates before (top) and after (bottom) crystallization. (e) Adapted with permission [34]. Copyright 2023, American Chemical Society. (f) Adapted with permission [18]. Copyright 2023, Wiley‐VCH GmbH.

Se and Te share an isomorphic phase diagram with complete solid solubility [28] (Figure 2c). The thermodynamically stable phase is the trigonal (γ) phase consisting of 1‐dimensional covalently bonded chains held together by van der Waals forces (Figure 2a). The γ phase of interest for PVs and PDs differs from the amorphous (*a*‐) Se and Te_x_Se_1‐x_ used in xerography and X‐Ray detectors.

Although the γ‐Se phase is the thermodynamically stable phase of Se, there are several metastable phases of Se consisting of fused Se rings that should not be ignored. Typically, these are Se_8_ rings in a monoclinic (*m*‐Se) structure (Figure 2b), but orthogonal Se_6_ or rhombohedral Se_7_ ring structures similarly exist. These phases become important when considering the conversion of *a*‐Se to γ‐Se. *a‐*Se has different structures depending on the processing method: melt‐quenched Se consists primarily of amorphous chain‐like Se, while vapor processed Se consists primarily of Se_8_ rings [29, 30, 31]. Lu et al. showed that these rings are highly unstable and readily undergo a thermally induced or photo‐induced ring opening reaction to become Se chains [31]. In practice, *a‐*Se films can be expected to contain a combination of disordered Se chains and Se ring monomers, though the processing method will have an impact on the ratio between these structures.

Understanding the complex interplay between Se rings and chains is important in conventional two‐step processed Se films where *a‐*Se is deposited by thermal evaporation (TE) and then annealed above the crystallization temperature of approximately 121°C. In practice, the anneal is typically done at a temperature between 200°C and the melting point of 221°C. Upon annealing, the phase transition results in a color change from red to dark gray (Figure 2e). Notably, the chain and ring‐like Se have different crystallization pathways (Figure 2d). Chain‐like *a‐*Se can crystallize directly in the γ‐Se phase in a single exothermic phase transition with an activation energy of approximately 1.28 kcal mol^−1^ [30]. Ring‐like *a‐*Se transitions first to the metastable *m*‐Se phase with a small activation energy (0.28 kcal mol^−1^), then the Se_8_ rings must undergo ring opening, polymerization, and ordering steps to form γ‐Se, overcoming a sizable energy barrier of ∼1.42 kcal mol^−1^ [30].

While Te similarly shares the γ phase, it has distinct differences from Se. Unlike Se, Te does not form metastable ring‐like phases. Even in the 2D limit where Te forms varied stacking arrangements, all phases consist of chain‐like Te [32] (Our focus in this review is on “bulk” thin films, where only γ‐Te forms. For further discussion on 2D tellurene and its various polymorphs we point readers to another review [32]). The crystallization process also changes as Te is added. The activation energy for crystallization decreases with increasing Te concentration [20], and pure Te crystallizes in the γ phase spontaneously at room temperature [33]. For this reason, Te‐rich compositions are often deposited on cryogenically cooled substrates (−80°C) to slow the crystallization and achieve larger grain sizes [33]. Photographs of a Te_0.7_Se_0.3_ film before and after annealing are shown in Figure 2f.

### 2.2 Optoelectronic Properties

#### 2.2.1 Bandgap, Carrier Mobility, and Light Absorption

**FIGURE 3:**
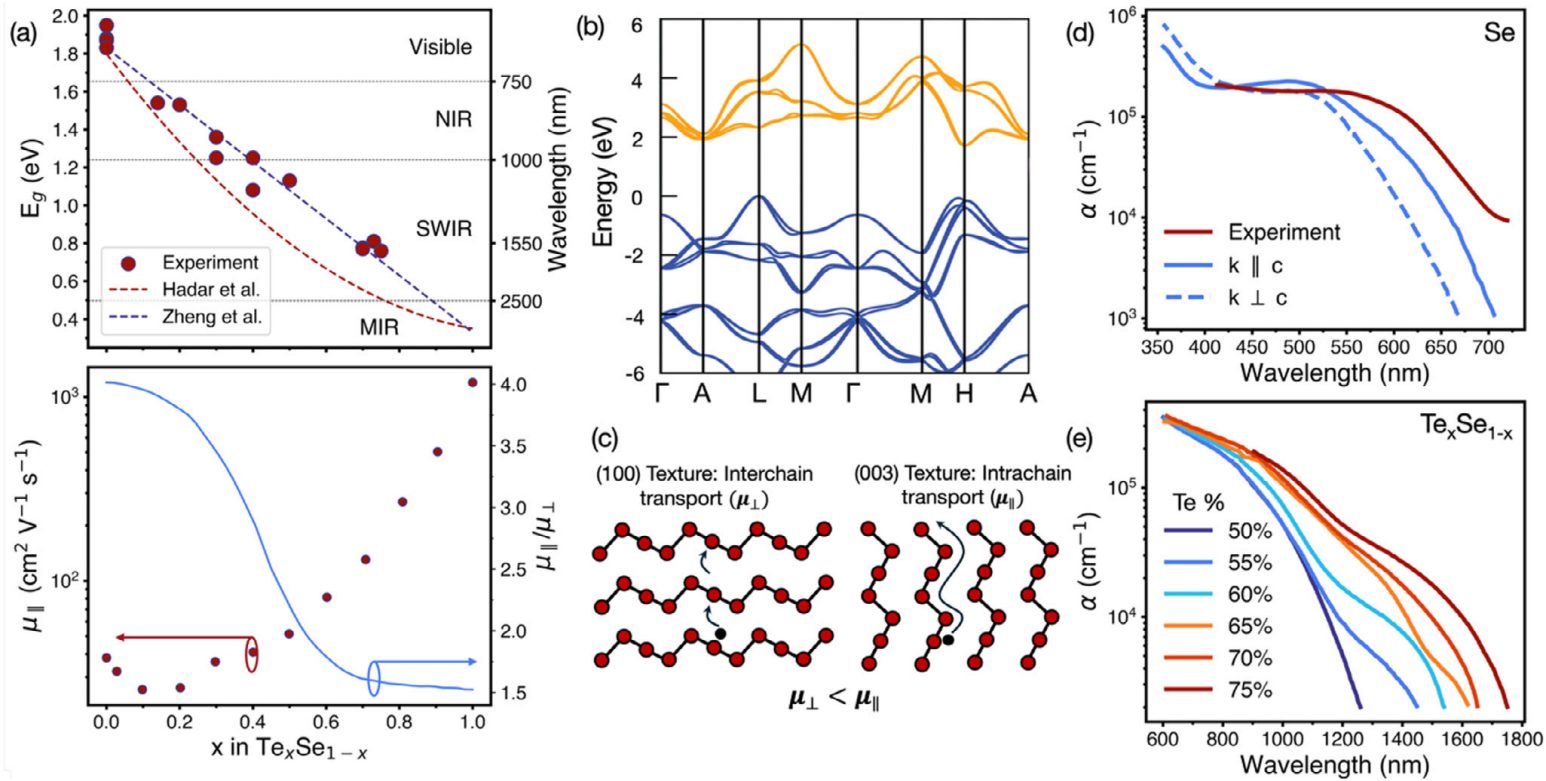
Optoelectronic properties of Se and Te_x_Se_1‐x_. Compositional dependence of **(a, top)** Bandgap energy (and corresponding wavelength, right axis) and **(a, bottom)** carrier mobility (left axis, red dots) and anisotropy (right, blue line). **(a, top)** includes experimentally extracted values of *E_g_* [14, 18, 22, 35, 36, 40, 41, 42, 43] (red dots) and two models for *E_g_* dependence on composition: a nonlinear model from Hadar et al. (red dashed line) and a linear model from Zheng et al. (blue dashed line). The regimes of the electromagnetic spectrum are listed to show the wide range of technologically relevant *E_g_*’s achievable. (b) DFT calculated band structure of pure Se. (c) Schematic showing the differences in interchain transport (μ_⊥_) and intrachain (μ_∥_) transport for γ‐Te_x_Se_1‐x_. In PV and PD device structures, (100) textures requires interchain transport while (003) texture allows intrachain transport, which has a higher mobility for all compositions. (d) Calculated absorption coefficient for pure Se with different directions of electromagnetic wave propagation relative to the crystal structure, replotted data adapted from [44]. An experimentally determined absorption coefficient for polycrystalline Se is included. (e) Absorption coefficient as a function of wavelength for a series of Te_x_Se_1‐x_ compositions relevant for SWIR PDs, replotted data adapted from [20]. (b) and (d) Adapted under the terms of the CC‐BY Creative Commons Attribution 3.0 International license (https://creativecommons.org/licenses/by/3.0) [44]. (e) Adapted with permission  [20]. Copyright 2023, Wiley‐VCH GmbH.

The bandgap (*E_g_*) tunability is the key feature of this materials system. Within the γ phase, *E_g_* can be tuned from the *E_g_* of Te to that of Se, covering a remarkable range of 0.3 eV to 1.9 eV (Figure 3a, top). This compositional tunability is not demonstrated by any other materials system and is achieved with only 2 elements within the same crystallographic phase. This *E_g_* window covers the mid infrared (MIR), short‐wave IR (SWIR), near IR (NIR), and visible range. There have been two models proposed for the value of *E_g_* as a function of Te concentration (x), a linear relation that follows Vegard's law [35] (dashed blue line) and a bowed nonlinear relationship [36] (dashed red line). The physical origin of bowing is not proposed, but observing nonlinearity is common in alloys with a variety of mechanisms, including structural distortion, band anti‐crossings, etc. [37, 38, 39] Experimental values tend to fall in the region between these curves [14, 18, 22, 35, 36, 40, 41, 42, 43], though Vegard's law tends to be accurate for the higher Te‐content alloys that are of interest for PDs. The band structure of Se is shown in Figure 3b [44]. The valence band maximum (VBM) is at the *L* point of the Brillouin zone, while the conduction band minimum (CBM) is at the *H* point, where the direct optical transition occurs. A local maximum in the valence band sits slightly offset from the *H* point along the *H‐K* direction, slightly lower than the absolute VBM. For pure Te, the shape of the band structure is similar, but the VBM is the point slightly shifted from the *H* point along *H‐K* [45]. Se and Te_x_Se_1‐x_ are therefore slightly indirect or “quasi‐direct” gap semiconductors. The optical *E_g_* is only ∼0.1 eV wider than the electronic *E_g_*, so absorption losses are minimal compared to other indirect gap semiconductors. The quasi‐direct gap, as well as optically active shallow defect states [46, 47], likely influence the *E_g_* extracted from Tauc plots. PL could be used to more accurately determine the optical bandgap while another method will be necessary to determine the indirect gap.

Te_x_Se_1‐x_ alloys of all compositions are p‐type semiconductors. Figure 3a, bottom shows the hole mobility parallel to chains (μ_∥_, red dots, left axis) and the ratio of the hole mobility along the parallel vs perpendicular directions (μ_∥_/μ_⊥_, blue line, right axis) [48]. Pure Se and Te have carrier mobilities primarily limited by phonon scattering [48]. However, Se has significantly stronger carrier‐phonon interactions that result in mobilities 1–2 orders of magnitude lower than Te. Te has a high hole mobility >1000 cm^2^ V^−1^ s^−1^ along the [001] direction, while pure Se has a significantly lower hole mobility of approximately 10–40 cm^2^ V^−1^ s^−1^ along the [001] direction [48, 49, 50]. The sum mobility extracted from transient photoconductivity measurements in Se single crystals is 40 cm^2^ V^−1^ s^−1^ along [001] and 17 cm^2^ V^−1^ s^−1^ along [100] [50]. For Te_x_Se_1‐x_, alloy scattering introduces a nonlinear dependence of hole mobility on composition. In general, the carrier mobility is higher for Te rich compositions, and the minimum carrier mobility is exhibited by Te_x_Se_1‐x_ compositions with 0.1< x < 0.2. The μ_∥_/μ_⊥_ ratio (Figure 3a, bottom) as a function of composition highlights the variation in anisotropy. Se has highly anisotropic transport, which is expected from the 1D crystal structure. Interestingly, despite the 1D crystal structure, strong inter‐chain interactions in Te result in more isotropic transport [51]. This reflects the anisotropic conductivity effective mass of electrons (m_e_*) and holes (m_h_*) for pure Se and Te, which are listed in Table 1. m_e_^*^ and m_h_* are 2.2 and 3.2 times heavier, respectively, in the [100] direction than the [001] direction for Se. Meanwhile, the ratio of *m*_[100]_ * to *m*_[001]_ * for pure Te is between 1.5 for electrons and 1.8 for holes (averaging between the upper and lower valence bands). Phonon limited mobilities scale with (m^*^)^−3/2^ for optical phonons and (m^*^)^−5/2^ for acoustic phonons, so effective mass anisotropy is amplified by phonon‐limited transport. The greater anisotropy in Se suggests that Se PVs should have crystalline texture as close to (001) as possible for optimal transport (Figure 3c). For Te, the lower carrier‐phonon coupling and more isotropic effective mass relaxes constraints, but (001) orientation would still be expected to exhibit better bulk transport properties.

**TABLE 1:** Tabulated anisotropic carrier effective mass for pure Se and Te. Values for m_e_* and m_h_* for Se are computationally determined values from ref [42]. Values for Te are experimentally measured values from ref. [52] (for m_e_*) and ref. [53] (for m_h_^*^), which are in good agreement with theory [45]. For m_h_* in Te, values are listed for the valence band maximum (*H_4_*) and the lower valence band (*H_5_*) split off by 110 meV due to spin‐orbit coupling [45].

	Se [100] (⊥)	Se [001] (∥)	Te [100] (⊥)	Te [001] (∥)
m_e_*/m_0_	0.39	0.18	0.104	0.070
m_h_*/m_0_	0.68	0.21	0.108 (*H_4_*), 0.256 (*H_5_*)	0.220 (*H_4_*), 0.039 (*H_5_*)

Figure 3d shows the calculated absorption coefficient, *ɑ*, for Se along different axes and the experimentally determined *ɑ* for a polycrystalline film [44]. *ɑ* > 10^5^ cm^−1^ for most of the above‐gap range shows that Se is a strong optical absorber. Figure 3e shows the experimental *ɑ* for Te_x_Se_1‐x_ with x varying from 0.5 to 0.75 [20]. All compositions within this range exhibit *ɑ* > 10^5^ cm^−1^ at wavelengths shorter than ∼900 nm, while compositions x ≥ 0.6 have *ɑ* > 10^4^ cm^−1^ at 1300 nm. We note that while *ɑ* for these materials is quite large, it does not reveal the true potential of these materials as optical absorbers. Considering *ɑ* alone is only relevant to Beer‐Lambert absorption and does not account for the extent to which light can be confined in a material. The power absorbed per unit volume in a material can be represented as:

(1) $$P_{abs}\left(x,\lambda \right)=\frac{1}{2}n\alpha \left(\lambda \right)c{\left|E\left(x\right)\right|}^{2}$$

where *n* is the real part of the refractive index, c is the speed of light in a vacuum, and |*E*(*x*)| is the electric field magnitude at some location x. *n* is relevant to electric field localization through optical cavity modes such as Fabry‐Pérot resonances or more advanced optical resonators. With the importance of *n* for light harvesting in mind, it is worth recognizing that Te has massive *n* > 5 in the NIR and Se has *n* > 3 in the visible range [54]. The values for alloys have not been reported but can be expected to be between these bounds. More briefly, Se and Te_x_Se_1‐x_ have excellent optical absorption properties that a simple absorption coefficient does not fully communicate.

#### 2.2.2 Defects and carrier dynamics

**FIGURE 4:**
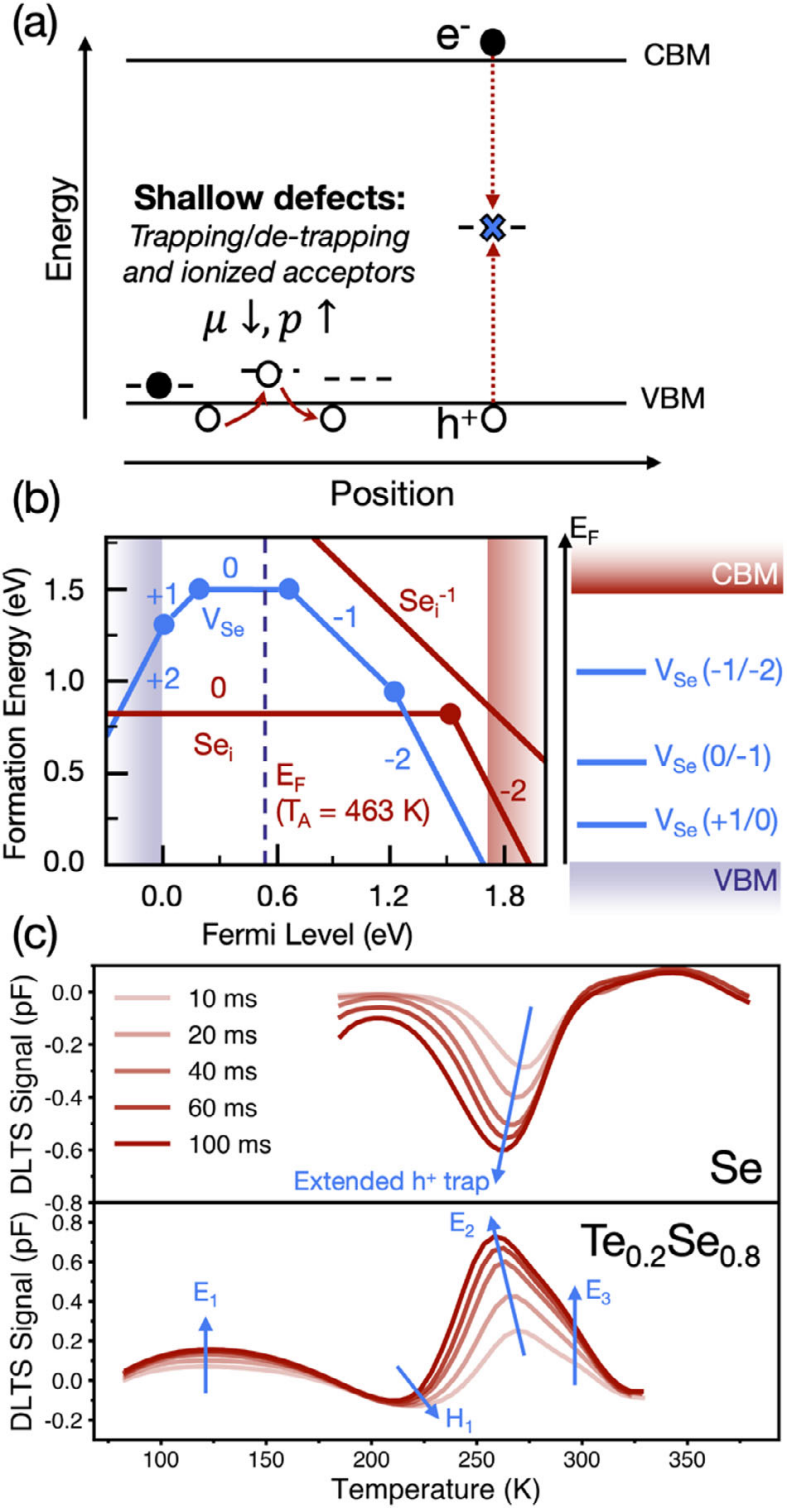
Defects in Se and Te_x_Se_1‐x_. (a) Schematic showing the effects of shallow and deep level defects on a semiconductor. Black circles represent electrons (e^−^) while white circles represent holes (h^+^). (b) Defect formation energy diagram for intrinsic defects in pure Se at the annealing temperature of 463K and corresponding charge transition levels for V_Se_ defects [44]. (c) Deep level transient spectroscopy (DLTS) signal for a Se (top) and Te_0.2_Se_0.8_ (bottom) PDs as a function of pulse width. Data is extracted and replotted from [65]. (b) Adapted with changes to fonts and colors under the terms of the CC‐BY Creative Commons Attribution 3.0 International license (https://creativecommons.org/licenses/by/3.0) [44]. (c) Adapted with permission [65]. Copyright 2024, American Physical Society.

Understanding the sources of nonradiative recombination in optoelectronic materials and devices is imperative to inform materials processing and enhance efficiency. These defects can be categorized as (i) deep‐level defects toward the center of the bandgap or (ii) shallow defects within several *k_B_T* of the conduction or valence band (Figure 4a). Deep level defects are generally considered more problematic for PVs as they result in greater Shockley‐Read‐Hall (SRH) recombination rates and, therefore, decrease the open‐circuit voltage (*V_oc_*) and fill factor (*FF*) of PVs. Shallow defects are not entirely benign, as these states introduce donor or acceptor states that impact doping levels and result in impurity scattering that decreases the carrier mobilities, thus decreasing carrier diffusion lengths.

Currently, the key open question in the field of Se PVs is identifying the defects that dominate nonradiative recombination in this system and the processing origins. Metal chalcogenide thin film PV absorbers such as CdTe, chalcopyrites, and kesterites are notoriously plagued by deep level intrinsic point defects [55, 56, 57, 58, 59, 60, 61], but the intrinsic point defects in Se appear to not account for the nonradiative recombination rates observed [62]. The *p‐p* antibonding nature of the valence band in Se results in shallow intrinsic point defects (V_Se_ and Se_i_) that can exhibit self‐healing due to low migration energies of bulk defects to the edge of chains [62]. The intrinsic defects in Se formed at an annealing temperature of 190 °C are shown in Figure 4b. Recent DFT studies by Kavanagh et al. and Moustafa et al. have suggested that intrinsic point defects (V_Se_ and Se_i_) in Se are benign in terms of recombination and extrinsic defects do not significantly contribute to p‐type doping [44, 63]. However, there are disagreements between these studies over the extent to which intrinsic defects impact doping and extrinsic defects impact recombination. Both studies find that extrinsic impurities (particularly pnictogens and halides) can introduce deep level defects that may act as recombination centers, and Kavanagh et al. show that F and Cl are present in Se films via secondary ion mass spectroscopy measurements. However, the presence of F and Cl do not account for the doping density [44]. It is worth considering if F and Cl defects contribute to recombination, even if not substantially affecting doping. Further, while the shallow intrinsic defects should not significantly impact the lifetime, these defects may impact carrier mobility and therefore diffusion length. Sn_Se_ extrinsic substitutional defects have also been suggested to introduce recombination centers that are most active under moderate to strong (10^16^–10^18^ cm^−3^) p‐type doping conditions [64]. The potential for extrinsic point defects to limit *V_oc_* depending on Se doping levels is worth further consideration, but studies on point defects generally suggest that extended defects are the dominant source of loss in Se.

The role of defects in Te_x_Se_1‐x_ alloys is also poorly understood. In addition to higher efficiency being desired, key challenges for Te_x_Se_1‐x_ PDs are the high dark current density and the relatively slow response times. Improving efficiency is likely related to eliminating deep level defects. High hole concentrations of 10^16^–10^17^ cm^−3^ for Te_0.7_Se_0.3_ are 2–4 orders of magnitude higher than the intrinsic carrier concentration expected for a ∼0.7 eV bandgap material. This is indicative of a relatively high density of shallow acceptor states that can be expected to (i) increase dark current due to the increased hole concentration, (ii) increase impurity scattering, decreasing the mobility and increasing transport times, and (iii) increase junction capacitance, increasing RC delay.

Deep level transient spectroscopy (DLTS) [65, 66] is a powerful device characterization technique to study the energy levels, concentration, and nature (extended vs. point, hole vs. electron) of deep level defects. DLTS studies of Se PVs have revealed that the deep trap states are most likely extended defects (Figure 4c, top) [65, 66], such as grain boundaries, interface states, regions with disordered chains, etc. Notably, some studies show signatures of only hole traps [29, 65] while others show both hole and electron traps [66], and the energies appear to change significantly with differences in materials processing [29]. This suggests the sensitivity of the defects in Se to changes in materials processing. DLTS on Se‐rich Te_x_Se_1‐x_ alloy PDs has similarly been performed (Figure 4c, bottom) that suggest more extended defects, as well as an electron trap state (labeled E_1_) that has behavior more representative of a point defect. More studies to understand the origins and effects of defects in Te_x_Se_1‐x_ are needed to guide rational and strategic development of this technology.

**FIGURE 5:**
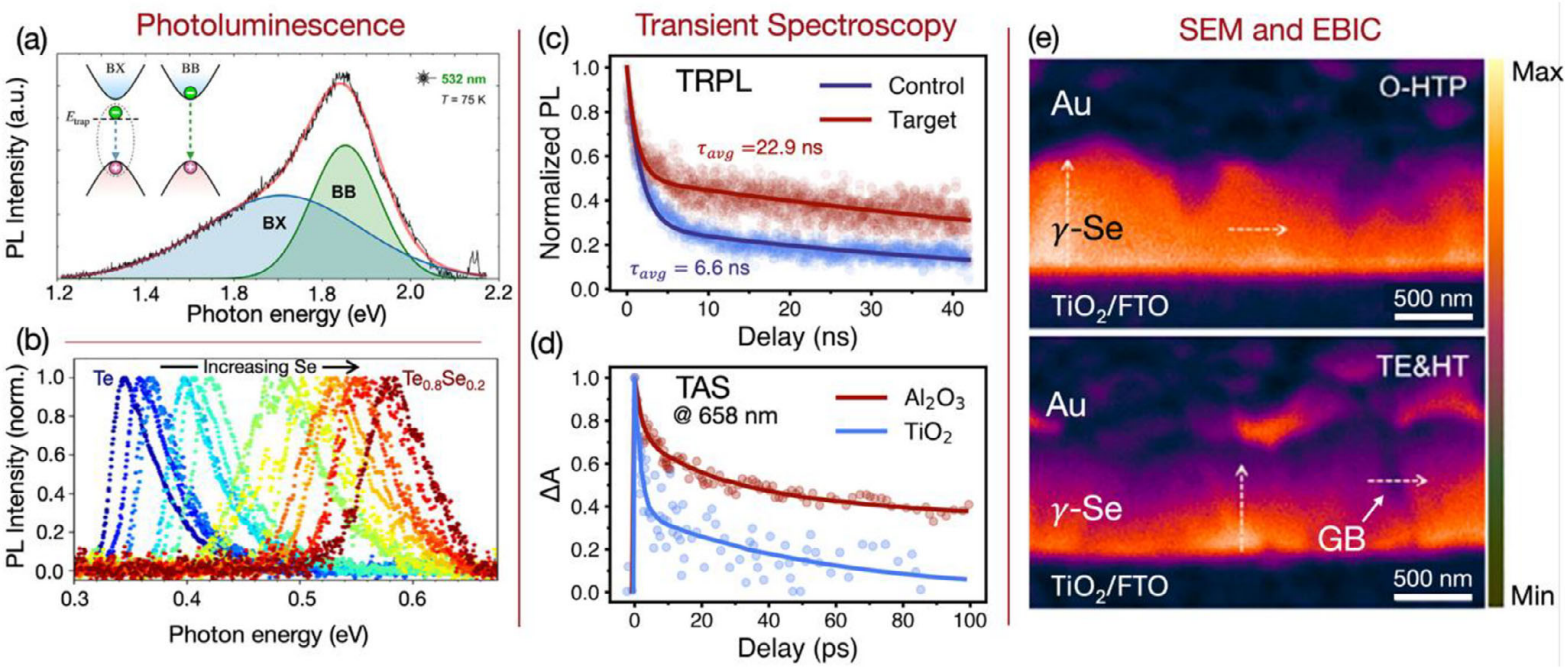
Optoelectronic Characterization Techniques (a) Photoluminescence (PL) spectrum collected at 75 K for Se, showing a peak corresponding to defect emission (BX) and a band‐to‐band transition (BB). (b) Normalized PL spectra for pure Te and Te‐rich alloy films of varying concentration, up to Te_0.8_Se_0.2_ [29]. (c) Time resolved PL (TRPL) for control and target samples showing an increase in the radiative lifetime, τ_avg_, to 22.9 ns. Plot is produced using the data provided in ref [69]. (d) Transient absorption spectroscopy (TAS) at 658 nm for Se on Al_2_O_3_ and TiO_2_ substrates, with data extracted from ref. [68] (e) Scanning electron microscopy (SEM) and overlaid electron beam induced current (EBIC) image of Se PVs with Se deposited by two methods (O‐HTP, top and TE&HT, bottom). Reduced current due to a grain boundary (GB) is seen in the bottom image. Figure (a) Adapted under the terms of the CC‐BY Creative Commons Attribution 4.0 International license (https://creativecommons.org/licenses/by/4.0) [46]. Copyright 2025, the Authors. (b) Adapted with permission [80]. Copyright 2025, American Chemical Society. (e) Adapted with permission [29]. Copyright 2025, Wiley‐VCH GmbH. Labels in (a, b and e) were altered for readability.

Nielsen et al. performed temperature‐dependent (T‐dependent) Raman and PL spectroscopy to study defects, disorder, and electron‐phonon coupling on Se films grown in 2 different labs [46]. They found that electron‐phonon coupling and grown‐in stress in films depended on processing conditions, and films that exhibited lower coupling strengths resulted in devices with higher *V_oc_*. We note that disordered Se chains have previously been suggested to introduce mid‐gap states [67] and could also introduce lattice strain. In addition to band edge PL, Se shows sub‐gap emission that is weakly T‐dependent and likely related to shallow defect states (Figure 5a). T‐dependent Raman and PL spectroscopy therefore offer a method to characterize both structural disorder and shallow defect states in Se. PL in Te_x_Se_1‐x_ films has also been demonstrated in compositions with x ≥ 0.8 (Figure 5b). These compositions do not appear to exhibit PL from shallow defect states, but it is not known how shallow defect emission evolves with composition at wider bandgap compositions. Nonetheless, the PL of Te_x_Se_1‐x_ suggests that the methods used by Nielsen et al. could be used to understand extended defects in Te_x_Se_1‐x_ as well.

Transient optical spectroscopy techniques such as transient absorption spectroscopy (TAS), time‐resolved photoluminescence spectroscopy (TRPL), and optical pump‐terahertz probe spectroscopy (OPTP) are valuable methods for studying carrier dynamics. TAS and OPTP results indicate fast carrier trapping/relaxation processes on the order of several ps, with longer decays on the scale of tens of ps to several ns [29, 42]. Notably, the initial decay in the TAS signal is dependent on the substrate, with TiO_2_ giving faster signal decay than Al_2_O_3_ [68] (Figure 5d), suggesting that the rapid initial decay could be the result of charge extraction. However, other more problematic mechanisms could be responsible: the soft lattice, relatively strong interactions between carriers and phonons, and quick OPTP and TAS decays make it worth considering whether intrinsic self‐trapping by lattice deformations could impact carrier dynamics in Se. TRPL (Figure 5c), however, gives radiative effective lifetimes >20 ns [69], as can be expected from the generally longer timescale of radiative processes [70]. More work to understand the lifetimes relevant to device operation and the factors that limit them is necessary.

We believe that the reports on carrier dynamics of Se studied by transient spectroscopy do not adequately account for confounding variables and do not provide convincing quantitative estimates of bulk carrier lifetimes. While comparisons between multiple samples with different processing or device layers remain qualitatively relevant, extracting an exponential fit and asserting that it represents the bulk lifetime in a thin film is an oversimplification of a highly complicated system in which transport, charge extraction, and surface recombination can influence the signal decay [71, 72, 73]. Without these considerations, the bulk lifetime and the photophysical processes that limit it may not be determined accurately, particularly for thinner films in which the interfaces play a large role. Moreover, greater care should be taken to account for anisotropy in optical pump‐THz probe measurements because, at normal incidence, the oscillating THz dipole is in‐plane and thus measures in‐plane photoconductivity. Meanwhile, the direction relevant to device transport is out‐of‐plane. Angle‐dependent OPTP measurements would enable contributions from the out‐of‐plane direction to be decoupled from the in‐plane direction, potentially yielding more accurate results for mobility and lifetime. The same argument can be made for time‐resolved microwave conductivity, which has been used to characterize Te_x_Se_1‐x_ and Se films [20, 74] and similarly probes transient photoconductivity with an oscillating electric dipole, but on longer (ns – µs) timescales than OPTP. Transient spectroscopy techniques are powerful tools to better understand the photophysics of Se and Te_x_Se_1‐x_, but extracting more meaningful and clear results will require better experimental design and more careful analysis.

To better connect structure with optoelectronic properties, characterization techniques that collect structural and optoelectronic information simultaneously and with high spatial resolution are valuable and, in our opinion, underutilized in this field. Shen et al. used electron beam induced current (EBIC) taken alongside scanning electron microscope (SEM) images to show an increased depletion region depth and reduced sensitivity to grain boundaries for films with improved (101) texture compared to a (100) textured control (Figure 5e) [29]. Atomic force microscopy (AFM) coupled with Kelvin probe force microscopy (KPFM) and/or conductive AFM (C‐AFM) is another useful technique that is valuable to probe grain boundaries and nanoscale inhomogeneities [75], among other applications. In the same work by Shen et al., they used AFM/KPFM to demonstrate decreased potential differences at grain boundaries in the (101) textured sample. This has similarly been performed for Te_x_Se_1‐x_ films annealed at different temperatures to reveal differences in grain boundary potential [76]. Further use of scanning probe methods and coupling C‐AFM and KPFM with near‐field optical spectroscopy or scanning photocurrent/photovoltage techniques could be valuable to study the influence of grain boundaries and other extended defects on optoelectronic properties [77, 78]. C‐AFM could also be valuable to identify possible shunting pathways in Te_x_Se_1‐x_ PDs [79].

For a compositionally simple system, the defect physics of Se and Te_x_Se_1‐x_ appear surprisingly complex and are poorly understood. There is strong evidence for both Se and Te_x_Se_1‐x_ that extended structural defects (grain boundaries, disordered chains or metastable Se_8_ rings, interfaces, stacking faults, etc.) introduce mid‐gap trap states that would be the most active nonradiative recombination sites. This would be more detrimental to Se PVs, decreasing the *V_oc_* and, with it, the *FF*, though *J_sc_* also becomes affected at high recombination levels. In Te_x_Se_1‐x_ PDs, the nonradiative recombination would influence responsivity (*R*) and the reverse saturation current, *J_0_*, potentially influencing *J_d_* depending on the dark current mechanism. The possibility of extrinsic defects from halides, pnictides, and group IV elements also merits further study to rule out these defects as prominent recombination centers. Shallow acceptor states, hypothesized to be attributable to intrinsic defects, would be expected to contribute to *p*‐type doping and a reduced *μ*, though the extent to which impurity scattering influences *μ* in this system has not been quantified. For Se PVs, the reduced *μ* would exert the most influence through the diffusion length. A lower *μ* could increase the response time in Te_x_Se_1‐x_ PDs, but the increased *p*‐type doping is a greater concern and would be reflected in or responsible for a large *J_d_*. Series resistance at the device level would degrade *FF* in Se PVs. A large *G_sh_* due to device level shunting would kill the *FF* and *V_oc_* in PVs and increase *J_d_* in PDs. Shunting does not appear to be a concern for Se but cannot yet be ruled out in Te_x_Se_1‐x_ PDs.

## 3 Application of Se PVs and Te_x_Se_1‐x_ PDs

### 3.1 Wide Bandgap Se PVs

#### 3.1.1 Indoor Photovoltaics (IPVs)

Roughly half of all IoT sensors are deployed indoors for applications that include healthcare, industry, commerce, and smart homes [81, 82, 83]. These sensors are often battery‐powered, resulting in increased demand for battery materials and electronic waste. The simultaneous growth of the IoT market and decrease in the power consumption of individual IoT nodes motivates powering these nodes with indoor photovoltaics that harvest ambient indoor lighting [12, 83, 84, 85]. The spectral irradiance of white LED (WLED) and fluorescent (FL) light sources is each concentrated in the visible portion of the spectrum. Consequently, the detailed balance limit of efficiency under indoor lighting puts the ideal bandgap for IPVs in the range of 1.8–1.9 eV and the maximum power conversion efficiency under indoor lighting (PCE_i_) of ∼54–57% [11].

The non‐ideal bandgap (1.1 eV) and high dark current make crystalline Si PVs unsuitable for this application. Hydrogenated amorphous‐Si (a‐Si:H) cells, with a wider bandgap of 1.7 eV, are the industry standard, but commercial a‐Si:H IPV modules tend to have a PCE_i_ <10% [12]. Research a‐Si:H IPV cells have improved to 29.9% (1000 lux) [86], but the spectroscopically limited maximum efficiency under indoor lighting (SLME_i_) for a‐Si:H is only 37% [12], limiting the extent to which this technology can improve.

Highly efficient LHP IPV minimodules with PCE_i_ over 43% have already been demonstrated [87]. LHPs are unquestionably a highly efficient, low‐cost material for optoelectronic applications, but the high toxicity of Pb renders these devices non‐RoHS compliant and becomes a serious concern in applications like IPV that operate near people. This raises questions about the suitability of LHPs for IPVs despite the high efficiencies, and it motivates the development of alternative materials with low toxicity.

Tin halide perovskites (THPs) are less toxic than their Pb counterparts, but the efficiency of these devices is much lower, with the best THP IPVs having ∼22.5% PCE_i_ [88]. Additionally, Sn^2+^ readily oxidizes to Sn^4+^, resulting in fast degradation and creating V_Sn_ defects that introduce p‐type self‐doping [15, 89, 90]. The dark conductivity of THP IPVs is therefore relatively high [91], which is a serious concern for IPVs considering the elevated importance of maintaining high shunt resistance under low illumination conditions [12]. This challenge and the closely related issue of THP stability present major obstacles. Other perovskite inspired materials (PIMs) have attracted attention, including heavy pnictogen (i.e., Sb^3+^, Bi^3+^) halides, which have achieved PCE_i_ > 10% [92]. However, carrier localization is common in this class of materials and may pose a fundamental limit [93]. There is space in this field for new low‐cost, non‐toxic, and efficient materials to emerge.

**FIGURE 6:**
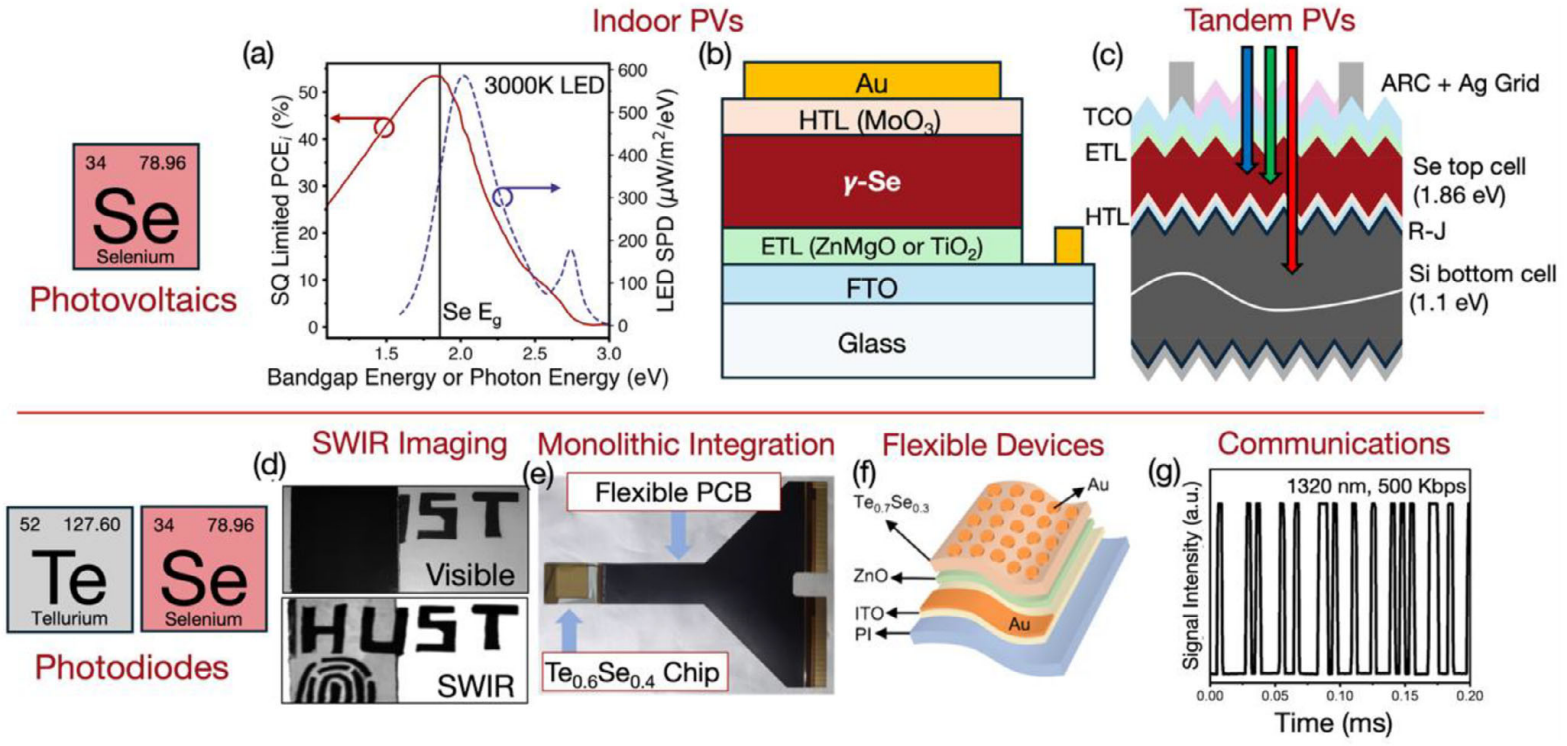
Applications of Se PVs and Te_x_Se_1‐x_ Photodiodes. (a) SQ‐limited PCE as a function of bandgap for 3000K correlated color temperature LED illumination (reproduced from ref. [11]) and the spectral power density (SPD) of a 1000 lux 3000K LED [95]. The Se bandgap (1.86 eV) is denoted with a black vertical line. (b) Common device structure of a Se IPV (and single‐junction Se solar cell) with a F:SnO_2_ (FTO) bottom electrode, electron transport layer (ETL), γ‐Se absorber, hole transport layer (HTL), and an Au top electrode. (c) Schematic of a possible Se‐Si monolithic tandem solar cell structure. R‐J stands for recombination junction, ARC stands for antireflective coating, and TCO stands for transparent conductive oxide. (d) Visual (top) and SWIR (bottom) imaging of the letters HUST partially covered by a Si wafer taken with Te_x_Se_1‐x_ PD array [18]. (e) Monolithic integration of a Te_x_Se_1‐x_ PD array on an amorphous Si flexible ROIC [19]. (f) Device structure schematic for a flexible Te_0.7_Se_0.3_ PD on a polyimide (PI) substrate [96]. (g) 500 Kbps optical communications signal operating at 1320 nm (telecom O‐band) using a Te_x_Se_1‐x_ PD [22]. (d) Adapted with permission [18]. Copyright 2023, Wiley‐VCH GmbH. (e) Adapted with permission [19]. Copyright 2024, American Chemical Society. (f) Adapted with permission [96]. Copyright 2024, American Chemical Society. (g) Adapted with permission [22]. Copyright 2025, American Chemical Society.

Se has an ideal bandgap (∼1.9 eV) for indoor light harvesting (Figure 6a), and its strong absorption results in a SLME_i_ of ∼51%. Se further offers the low‐temperature processing and point defect tolerance of perovskites combined with the stability and low toxicity of metal chalcogenides, making it a quite interesting system for IPV. Se was first utilized for indoor light harvesting in 1982 by Ito et al., whose 4.6% PCE (AM1.5G) CdSe/Se heterojunction PV achieved 11.6% efficiency under 400 lux FL light [3]. Since then, PCE_i_ for Se IPVs has exceeded 20% [14, 41]. Compared with other inorganic and hybrid materials, Se appears to offer the best combination of performance, non‐toxicity, cost, and stability, making it among the most promising materials to realize IPVs at scale. For more detailed discussion of the potential and current state of the field of IPVs, we point the interested reader to other reviews [12, 83, 85].

A typical structure for a Se IPV is shown in Figure 6b. Se PVs are made in a superstrate n‐i‐p configuration with a transparent conductive oxide (TCO, typically FTO) bottom electrode, an electron transport layer (ETL, typically Zn_1‐x_Mg_x_O (ZMO) or TiO_2_), the Se absorber, a hole transport layer (HTL, typically MoO_x_), and a top electrode (typically Au). Device and transport layer engineering are discussed in section 5.2.

#### 3.1.2 Tandem Solar Cells

Wide bandgap Se PVs could be used as a top cell in a Se‐silicon tandem solar cell, with PCE of 35–40% potentially achievable with current Si solar cell technology [10]. For 4 terminal (4T) Se‐Si PVs, Se would need to reach only ∼14% PCE to boost Si to 30% efficiency. The only demonstration of a Se/Si TSC was by Nielsen et al., who made monolithic 2‐terminal devices [94]. While the *V_oc_* reached an impressive 1.68 eV, showcasing the potential promise, the short‐circuit current density was <1 mA/cm^2^. The authors identified the low EQE of the Se cell when illuminated through the HTL side as a limiting factor. We would therefore envision that a monolithic Se‐Si TSC would need a structure like that in Figure 6c, in which the Se is deposited on an HTL layer with an interconnecting layer on a textured Si solar cell, then the ETL, TCO, and antireflective coating are deposited sequentially on top of the Se film.

As Se solar cells continue to improve, it will be interesting to further develop tandem cells. The low cost of Se suggests that Se‐Si tandems could potentially enable a lower cost per watt, but a full technoeconomic analysis will be needed as Se develops. For 2J cells with Si, it will be necessary to either (i) identify how to invert the device structure to ensure illumination is through the ETL or (ii) develop 4T devices to eliminate constraints imposed by current‐matching and device structure. Another unexplored approach is the use of Se in all‐thin film devices with chalcogenides, perovskites, or organic cells. Se could potentially replace mixed I‐Br wide bandgap perovskite absorbers in 3J devices for improved stability. Se as an absorber for TSCs is a long‐term goal that should merit more consideration as the PCE of single junction Se PVs improves.

### 3.2 Te_x_Se_1‐x_ IR Photodiodes

#### 3.2.1 Photodiode Performance Metrics

Photodetectors convert an optical signal to an electrical signal. We will briefly define key photodetector performance metrics, as these parameters are less obvious than those for PVs, which operate at steady state and have key values that can be directly extracted from J‐V curves under a given illumination.

The responsivity (*R*) is a function of wavelength, *λ*, and is the photocurrent *I_ph_* divided by the input optical power, *P_opt_*. *R* effectively measures the efficiency of a photodetector and is closely related to the external quantum efficiency (EQE):

(2) $$R\;\left(\lambda \right)=\frac{I_{ph}\left(\lambda \right)-I_{Dark}}{P_{opt}}=\frac{q\lambda }{hc}EQE\left(\lambda \right)$$

The specific detectivity, D^*^, relates *R* to the frequency‐dependent signal noise density, *i_n_* (*f*), and the square root of the device active area, A:

(3) $$D^{*}\;\left(\lambda ,f\right)=\frac{R\left(\lambda \right)\sqrt{A}}{i_{n}\left(f\right)}$$

D^*^ is directly related to the dark current through *R* and shot noise contributions to *i_n_* (*f*). For shot noise‐limited D^*^ dominated by dark current density (*J_d_* = *I_Dark_*/*A*) contributions:

(4) $$D^{*}\left(\lambda ,f\right)=\frac{R\left(\lambda \right)}{\sqrt{2qJ_{d}}}$$

The range over which *R* is linear with respect to the light intensity is given by the linear dynamic range (LDR), reported in dB. The LDR is typically limited on the lower end by the noise effective power and can be limited by Auger recombination and forms of bimolecular recombination at high intensities. For light intensities *L_upper_* and *L_lower_* at the upper and lower limits of the linear regime, respectively, the LDR is:

(5) $$LDR\left(\lambda \right)=20\log\left(\frac{L_{upper}}{L_{lower}}\right)$$

Finally, the speed of a photodetector is characterized by the rise/fall times of photocurrent transients (τ*_r_* and τ*_fall_*) and the light modulation frequency at which the device can operate. The latter is defined by the −3dB bandwidth, that is, the light modulation frequency at which the photocurrent signal drops by −3dB (approximately half). The response speed is influenced by the carrier transport time from generation to collection and the RC delay introduced by the series resistance and junction (and electrode) capacitance.

Compared to photoconductors and phototransistors, PDs typically offer faster response times, low dark currents, and low noise that enables superior performance at low light intensities and large LDRs [97, 98]. PDs are therefore typically the photodetector of choice for imaging and communications.

#### 3.2.2 SWIR Photodiodes

PDs in the SWIR (loosely defined as the range from 0.9–2.5 µm) are commonly used for applications including telecommunications [99], night vision [100, 101], industrial quality inspection/control [100], security/surveillance [102, 103], etc. In addition to these well‐established applications, rapid development in the fields of light detection and ranging (LiDAR), augmented/virtual reality (XR), and biomedical imaging increase demand for low‐cost SWIR imaging.

LiDAR is commonly used for ranging and 3D imaging applications including metrology and environmental monitoring [104, 105, 106], but it is increasingly important due to its use in machine vision for emerging applications including robotics and autonomous vehicles [107, 108, 109, 110, 111]. LiDAR conventionally operates between 900–1000 nm or at the Nd:YAG laser line (1064 nm), but SWIR imaging in the range of 1300–1600 nm is being pursued for LiDAR and XR due to reduced atmospheric scattering and improved laser eye‐safety [112, 113, 114, 115]. Similarly, SWIR imaging is increasingly important for noninvasive in vivo fluorescent biomedical imaging, enabling reduced scattering and attenuation compared to visible and NIR wavelengths for high resolution imaging of brain tissue, tumors, blood vessels, etc. [116, 117, 118, 119]. The rapid growth of these fields and the increasing number of sensors deployed motivate the development of lower‐cost and low‐power SWIR PDs. The demand for wearable devices for point‐of‐care biomedical imaging additionally motivates the development of flexible SWIR PDs [120].

Current materials for PDs in this spectral range struggle with simultaneously achieving low cost, high performance, light weight and compatibility with flexible substrates, and low toxicity. III‐V materials are expensive, incompatible with CMOS back end of line (BEOL) processing, and not readily compatible with applications requiring flexible or lightweight devices. Thin film SWIR PDs based on PbS and HgTe colloidal quantum dots (CQDs) have been developed over the past 2 decades as high‐performance, low‐cost options that can be easily integrated with flexible substrates and read out integrated circuits (ROICs) [121, 122, 123, 124]. However, Pb and Hg are highly toxic. While there are ongoing research efforts to develop non‐toxic SWIR CQD PDs based on Ag_2_Te [112, 125, 126] and In(As, Sb) [127, 128, 129], these technologies contain relatively expensive metals and do not yet match the performance of PbS or HgTe CQD PDs. Therefore, materials innovation for thin film SWIR PDs with low cost, low toxicity, and high efficiency is needed.

As discussed in Section 2.1, Te_x_Se_1‐x_ has excellent bandgap tunability that covers SWIR wavelengths, high absorbance, and high carrier mobilities. As discussed in Section 3.3, it offers low toxicity, low‐temperature processing <260°C enabling monolithic integration with ROICs (Figure 6e) [19] or on flexible substrates (Figure 6f) [96], and good stability. Compared to emerging heavy metal‐free CQDs, Te_x_Se_1‐x_ has significantly lower material costs: Se ($23/kg) and Te ($80/kg) are significantly less expensive than Ag ($752/kg) and In ($395/kg) for pure elements [130].

Tan et al. demonstrated back‐gated Te_x_Se_1‐x_ photoconductive detectors [131] for the first time in 2020. Fu et al. developed the first Te_x_Se_1‐x_ thin‐film SWIR photodiode in 2023 (Figure 6d), demonstrating good *R* of 0.11 A/W at 1300 nm, a −3 dB bandwidth of 116 kHz, and sub‐1 µs response times [18]. Te_x_Se_1‐x_ PDs have since achieved *R* of ∼0.5 A/W at 0V applied bias at a wavelength of 1320 nm [22], superior to Ag_2_Te and In‐V CQD devices. D^*^ values > 10^11^ Jones at 1320 nm and >10^12^ Jones at 980 nm are comparable to the best CQD PDs. The sub‐µs response times have been leveraged for optical communications at the telecom wavelength of 1320 nm at a speed of 500 Kbps (Figure 6g) [22]. One can therefore expect that Te_x_Se_1‐x_ is a promising material for low‐cost and low‐toxicity SWIR PDs without sacrificing performance.

### 3.3 Practical Considerations

The practicality of materials for widespread use in optoelectronics is largely dependent on the toxicity and abundance of the constituent elements. For sustainable development, it is further crucial to consider environmental risks, energy intensity of processing, and designing for circularity. In this section, we consider the practicality of Se IPVs, Se for tandem solar cells, and Te_x_Se_1‐x_ for PDs.

#### 3.3.1 Toxicity

First, we consider the toxicological effects of Se and Te on humans. It is imperative to recognize that Se and Te have vastly different levels of biological activity and toxicity depending on the form of Se and Te, with oxyanions being far more hazardous than elemental forms. Selenium is an essential nutrient and is non‐toxic in low doses (recommended upper intake level of 255 µg/day), but oral consumption of selenite above 850 µg per day can lead to chronic selenosis, with potential for adverse effects as low as 330 µg per day [132]. Te is nonessential, and Te oxyanions (tellurites and tellurates) can be highly toxic [133]. The elemental forms of Se and Te that are discussed in this review have significantly lower biological activity than selenite and tellurite salts [134]. Elemental Se and Te have median lethal doses of 6700 and 5000 mg kg^−1^, respectively, compared to 12.7 mg kg^−1^ (oral) for selenite and 12.5 mg kg^−1^ for tellurite [133, 135, 136]. Te oxidizes readily to TeO_2_, which has a comparable median lethal dose to Te of 2000 mg kg^−1^ [133]. Selenite and tellurite salts do not easily form from elemental Se/Te, and the insolubility of elemental Se and Te in water and other common solvents reduces exposure risks, environmental hazards, and the physiological effects of exposure to these elemental forms. A 10×10 cm^2^ PV with a 500 nm thick Se absorber contains ∼24 mg of Se. This device would then be encapsulated between impermeable layers of glass and/or plastics with little opportunity for exposure. Te_x_Se_1‐x_ PDs will have an even smaller active area and therefore contain less material. Considering the low mass of chalcogenides used in devices, the low toxicity of elemental chalcogenides, and the low risk of exposure, elemental chalcogenide optoelectronics do not appear to present a hazard to consumers.

We further consider worker safety during industrial processing; the recommended exposure limit (REL) for a typical workday is 0.1 mg/m^3^ for Te and 0.2 mg/m^3^ for Se. As a comparison, metallic copper, which is widely used in industry, also has an REL of 0.1 mg/m^3^ [137]. We therefore conclude that elemental Se and Te_x_Se_1‐x_ are safe materials with low exposure risk to consumers and – provided occupational health and safety measures are followed – industrial workers.

Concerns of toxicity also should include consideration of ecological effects. For solar cells and waste that ends up in landfills, it is important to consider the possibility of leaching if encapsulation is damaged. While this has not been studied, to our knowledge, for elemental Se and Te_x_Se_1‐x_, these studies have been performed on Cu(In, Ga)Se_2_ (CIGS) and CdTe thin film photovoltaics [138, 139, 140, 141]. The leaching rates of Se and Te are low and do not increase the Se or Te concentration in water above levels set by regulatory agencies, though the bioaccumulation of Se [142] still motivates minimizing any potential leaching. Notably, long‐term leaching in landfill situations could pose a greater risk [141], encouraging recycling of optoelectronics instead of disposal in a landfill. It is unclear how the findings for these selenide and telluride solar cells translate to elemental Se and Te_x_Se_1‐x_ optoelectronics, so we encourage further studies on these materials directly. As it stands, it appears that proper disposal and recycling of Se and Te_x_Se_1‐x_ optoelectronics should minimize ecological hazards.

#### 3.3.2 Operational Stability

Operational stability is a challenge in many emerging thin film optoelectronic materials, most infamously LHPs. However, this challenge does not appear to plague Se and Te_x_Se_1‐x_ optoelectronics. Se PVs have repeatedly demonstrated negligible performance losses for 1000 h at maximum power point tracking under 1 sun illumination in ambient conditions without encapsulation [67, 69, 143]. This stems from the robustness of Se against oxidation [14] and its covalent bonding, rendering it stable against oxygen and water. Thermal cycling and other accelerated aging tests will need to be performed for potential Se tandem PVs, but this is not an immediate hurdle. Se certainly exhibits sufficient stability for IPVs.

While Te oxidizes more readily than Se, He et al. demonstrated Te_x_Se_1‐x_ PDs that only lost 5% of their initial *R* after aging at 85°C in air for 106 h and exhibited no performance loss after 24 h immersion in water [96]. Encapsulation with a Si_3_N_4_ barrier appears sufficient to prevent degradation in ambient conditions after 100 h of aging at 120°C in air [18]. These results suggest that Te_x_Se_1‐x_ is stable enough against common degradation sources for commercially practical applications.

#### 3.3.3 Sustainability

Earth abundance of materials is important to consider for solar and indoor light harvesting applications where the amount of power generation is proportional to the area of the cells. This is particularly the case for solar energy, which targets multi‐terawatt (TW) global capacity. We will therefore consider whether Se is sufficiently abundant for IPV modules and tandem solar cells at scale.

A current conservative estimate of global Se reserves is 95 metric kilotons [130]. Se is generally isolated as a byproduct of mining Cu and Zn, and the estimates of global reserves do not account for the potential of using “green” bio‐renewable sources of Se [144]. Sourcing Se from bio‐renewable sources is generally not pursued given the low demand for the element, but the possibility of it is a potential benefit that could be leveraged if desired. For commercial IPVs, an upper estimate of demand would be 10^6^ m^2^ of devices per year [83]. For Se IPVs with a 500 nm thick Se absorber, this translates to a Se consumption of ∼2405 kg, <0.1% of the 3600 metric tons of Se produced each year and 0.0025% of current global Se reserves [130]. We can therefore assert that, despite being a relatively scarce element, Se is more than sufficiently abundant for commercial IPVs at scale, especially with proper recycling. However, for Se‐Si tandem solar cells, scaling to multi‐TW global capacity presents challenges. If, quite optimistically, we assume that Se‐Si tandem PVs can reach 35% PCE, it would require ∼3×10^9^ m^2^ device area for 1 TW of electricity. With a 500 nm thick Se absorber, this equates to 7215 metric tons of Se, 7.6% of current global reserves and twice the amount of Se produced each year. Assuming global Se production remains constant at 3600 metric tons per year until 2050, this would mean that 500 nm thick Se/Si tandems could account for a maximum of 23.8 TW capacity, nearly 1/3 of the 75 TW capacity target by 2050 [145]. In short, the abundance of Se is sufficient for Se/Si tandem PVs to contribute a significant portion of the capacity needed for the energy transition to a global net zero situation. Se/Si tandem PVs would not be reasonably expected to lead the growth of solar energy given the current maturity level of Se PVs, but the relative scarcity of Se would not restrict Se/Si tandem PVs from contributing a large fraction of the necessary global PV capacity if/when performance becomes competitive with other technologies. However, increased Se production and decreased absorber film thickness would be needed to avoid depletion of global Se reserves. Analyzing the feasibility of increasing Se production should be considered in the event that Se/Si tandem PVs are commercialized, but this analysis is beyond the scope of this review.

The market size for SWIR imaging systems is predicted to exceed $1B in the next decade [146, 147]. For Te_0.7_Se_0.3_ PD array SWIR cameras with an active layer thickness of 500 nm, lateral dimensions of 1 cm^2^, and a film density of 5.87 g/cm^3^, the Te consumption required to make 1 billion devices is approximately 232 kg of Te, 0.0006% of global Te reserves [130]. Despite Te being a scarce element, the reduced scaling requirements of PDs compared to utility‐scale solar means that Te_x_Se_1‐x_ SWIR imaging systems could be commercialized without excessive Te consumption.

A final consideration of sustainability is designing for a circular economy. One should therefore consider absorber materials for both PVs and PDs that can be easily recycled. The vapor pressure of Se and Te are significantly higher than the vapor pressure of other typically used materials in the device stack (metal oxides and Ag or Au). Consequently, solvent‐free recycling of Se and Te optoelectronics can be accomplished at relatively low temperatures, and Wang et al. demonstrated Se IPVs with a 98% recycling yield and minimal change in the performance of recycled devices [148].

In summary of Section 3.3, (i) elemental Se and Te are safe materials with promising operational stability; (ii) Se has sufficient abundance for IPVs at scale and, potentially, tandem PVs at scale pending changes in absorber layer thickness and Se production; and (iii) recyclability will reduce waste, materials consumption, energy costs associated with extracting Se and Te from metal mining waste, and ecological effects that may arise due to bioaccumulation of these materials. A full life cycle analysis for each class of device (IPV, PV, PD) will be valuable, but this is beyond the scope of this review.

## 4 Processing Methods

### 4.1 Vapor Phase

Se and Te have low melting points and high vapor pressures. This is particularly the case for Se. Facile evaporation of elemental materials without requiring a chemical reaction of any sort makes sublimation‐based methods the most common route to depositing Se and Te_x_Se_1‐x_.

#### 4.1.1 Thermal Evaporation (TE)

Elemental chalcogenide thin films are typically deposited by TE. The conventional process to deposit Se involves TE before post‐annealing to crystallize the film in the desired trigonal phase. Note that we discuss the annealing process and recent advances in more detail in a later section. Similarly, Te_x_Se_1‐x_ is typically deposited by TE using a single‐source precursor prepared by heating mixtures of Te and Se powder in a sealed ampoule for extended periods of time. The annealing temperature for Te_x_Se_1‐x_ is typically higher (∼250°C) and conducted in an inert environment to avoid oxidation of Te.

For pure Se deposited by TE, it is conventional to first evaporate a 0.5–2.5 nm thick Te adhesion layer. While this interlayer was first introduced decades ago [4], its effects were not elucidated until recently by Yan et al. [149]. The TiO_2_ electron transport layer (ETL) is a highly inert surface, and Se has a high oxidative resistance. Consequently, Se does not easily bond with surface Ti or O atoms, resulting in poor adhesion and defects at the interface from both Se and TiO_2_ (i.e., dangling bonds, oxygen vacancies, etc.). Yan et al. showed that Te can enter Te^4+^ states readily, bonding with undercoordinated oxygen sites and “bridging” the Se to the metal oxide ETL. Instead of a uniform layer, the Te interlayer forms small islands. Depth‐dependent XPS measurements on pure Te films presented in this work exhibited Te° further from the ETL interface with Te^4+^ at the interface. We hypothesize that this holds true for the Te islands. By increasing the nominal Te thickness, the surface coverage of the Te islands increases. They demonstrated that by optimizing the surface coverage from ∼6% (0.5 nm nominal Te thickness) to ∼70% (2.5 nm nominal thickness), the interfacial defect density could be decreased, leading to an average PCE_i_ increasing from 11.4% to 14.1%.

**FIGURE 7:**
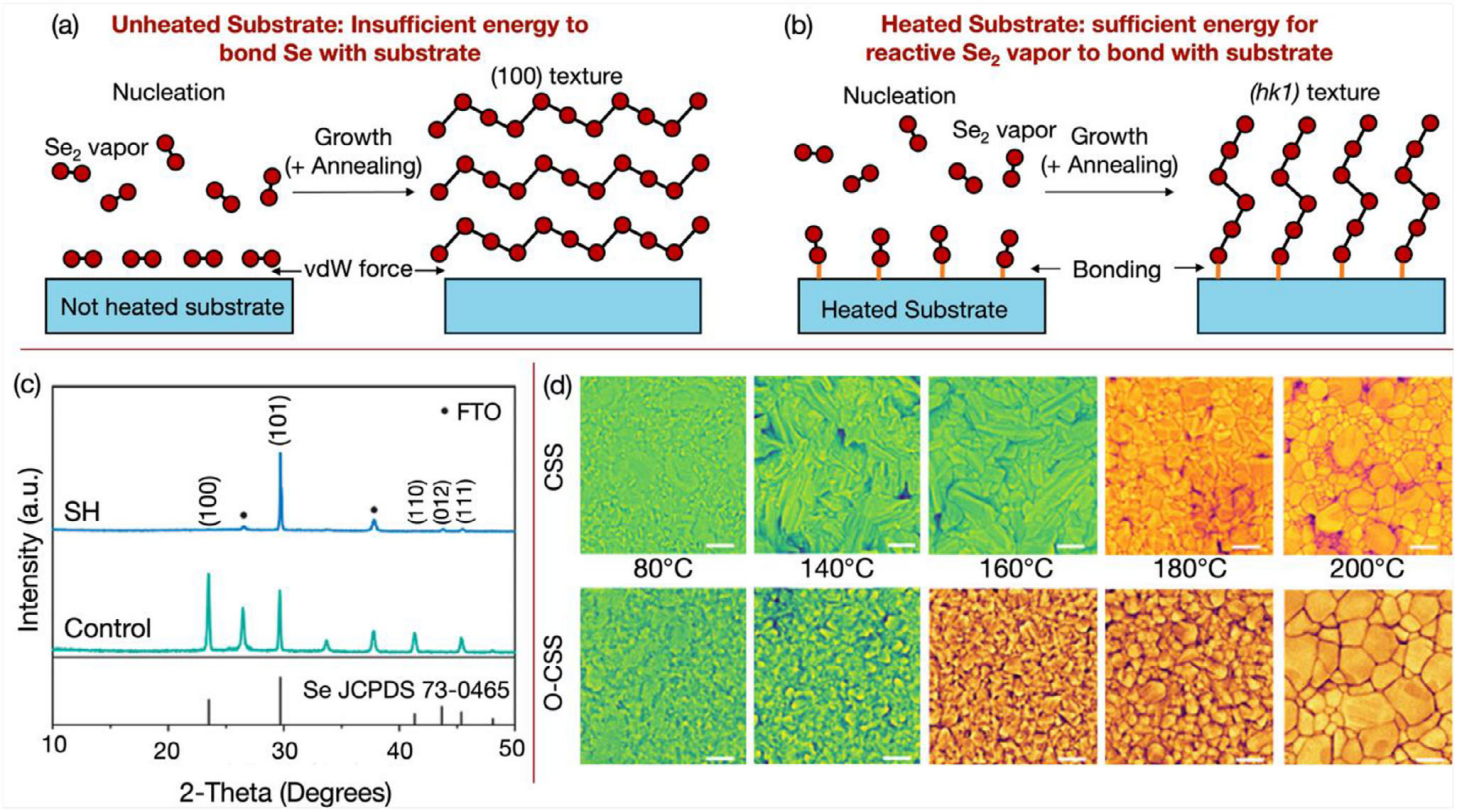
Vapor phase deposition. (a, b) Schematics showing the effects of substrate heating during vapor phase deposition without (a) and with (b) substrate heating. Without substrate heating, neither the Se_2_ molecules resulting from Se evaporation nor the solid Se films have sufficient energy to bond with the substrate, resulting in van der Waals (vdW) interactions and (100) orientation. During vapor deposition with substrate heating, the Se_2_ vapor reacts with the substrate, nucleating standing chains. (c) XRD spectra of Se films deposited by TE with (SH) and without (Control) substrate heating [143]. (d) False‐colored SEM images for CSS‐deposited Se films with (O‐CSS, bottom) and without (CSS, top) oxygen flow for substrate temperatures varying from 80°C to 200°C [29]. The green color is used to designate (100) textured films, while the orange texture is used to show (101) textured films. Oxygen flow and increased temperature enhance grain size and (101) texture in CSS‐deposited Se films. Oxygen flow also reduces the presence of pinholes (dark spots) in the films. (c) Adapted with permission [143]. Copyright 2024, Wiley‐VCH GmbH. (d) Adapted with permission [29]. Copyright 2025, Wiley‐VCH GmbH.

In another quasi‐1D vdW material, Sb_2_Se_3_, it is well established that orientation of the film out‐of‐plane with (001) or (hk1) texture is highly desirable because (i) the carrier mobility along directions of covalent bonding in vdW materials is higher than in directions of vdW forces and (ii) grain boundaries in (001) oriented quasi‐vdW materials are self‐passivated and therefore not a source of recombination [150]. This holds true for Se films [151], and the motivations for achieving (001) aligned Se films are clear based on the anisotropic transport discussed in Section 2.2. However, most Se films exhibit a mix of (100) and (101) orientations, often with (100) texture being dominant in thermally evaporated and annealed films [67, 69, 152]. Hadar et al. showed that TE of Se on a heated substrate led to greater (101) texture at the cost of film morphology [152]. Liu et al. demonstrated that by reducing the evaporation rate, uniform Se films could be thermally evaporated on a substrate held at 110°C, resulting in strong (101) texture in these films (Figure 7c) [143]. The improved texture enabled by substrate temperature is related to the interfacial bonding, as shown schematically in Figure 7a,b. Se_n_ chains have high bonding energies with metal oxide substrates, so they tend to minimize their surface energy through vdW interactions. Instead, the Se_2_ vapor produced during Se sublimation is more reactive, and substrate heating during vapor phase deposition can provide sufficient thermal energy for Se_2_ to react with the heated substrate and form covalent bonds. This bonding leads to the nucleation of vertically oriented Se chains. The vertical orientation achieved by Liu et al. was correlated with enhanced electron mobilities and a 1% absolute increase in PCE under AM1.5G lighting. They demonstrated that this approach could be extended to Te, suggesting that it should be viable across the entire Se‐Te binary system. To our knowledge, this approach has not been applied to Te_x_Se_1‐x_ films for PDs.

#### 4.1.2 Alternative Sublimation Techniques

As discussed in Section 2.1, the thermodynamic landscape in 2‐step TE + crystallization must contend with metastable Se phases. Alternative vapor‐based approaches that can directly deposit the γ‐Se phase with higher throughput and lower vacuum may be more desirable. Additionally, the relatively low throughput and capital costs associated with high vacuum requirements make TE less convenient for commercialization than other vapor phase approaches.

Close‐spaced sublimation (CSS) is a commonly used technique to deposit chalcogenide and perovskite thin film absorbers for PV with high throughput and low cost [153, 154, 155, 156]. In this process, a source material is rapidly sublimated while a substrate is spaced several mm away. The substrate temperature is independently heated to a temperature below that of the source material. This process is highly attractive for Se considering the high vapor pressure and lack of concerns regarding stoichiometry given the elemental nature. Shen et al. used an oxygen‐assisted close‐spaced sublimation (O‐CSS) technique, in which oxygen flow was introduced to the chamber during a CSS process, to overcome thermodynamic restraints on crystallization and achieve highly crystalline films with strong (101) texture [29]. The authors varied the substrate temperature from 80 to 200°C with a fixed source temperature of 210°C. Much like TE, increasing the substrate temperature led to a shift from (100) to (101) oriented films and larger grain sizes. The authors additionally found that introducing O_2_ gas flow significantly increased the grain size and eliminated pinholes (Figure 7d) without oxidizing Se or introducing oxygen in the resulting final films. Devices from O‐CSS films were compared against control devices made using a 2‐step TE process, which had (100) texture. The O‐CSS devices had higher carrier mobilities, lower defect densities, and longer carrier lifetimes. As a result, the average *V_oc_* increased from 0.79 V to 0.91 V, and the average PCE increased by almost 2%. This 120 mV increase in the *V_oc_* is, to our knowledge, the largest increase in *V_oc_* from engineering the Se absorber itself. The best device exhibited a PCE of 7.55% under AM1.5G lighting and 17.36% PCE_i_. These results suggest that O‐CSS is a promising approach to processing Se PVs.

Vapor transport deposition (VTD) is another sublimation‐based method that does not require high vacuum. In VTD, a source material and the substrate are physically separated in a horizontal tube furnace and held at different temperatures. A carrier gas, typically Ar, is saturated with the sublimated material and deposits it on a substrate held at a lower temperature [157]. Like CSS, VTD has been used in the deposition of chalcogenide and perovskite thin film absorbers for PV applications [157, 158, 159, 160, 161].

Caño et al. used VTD to make Se films for PV, and they explored the structure and device properties as a function of substrate temperature [162]. Substrates held at the optimal range of 112°C–120°C exhibited (003) textured films that resulted in the best device performance. From the works by Liu, Shen, and Caño, it is clear that vertically oriented (101) or (003) textured films are desired, and controlling substrate temperature is key in achieving this. Notably, in both the O‐CSS and VTD experiments with Se, adhesion to the substrate required thermally evaporating a Te interlayer prior to Se deposition. Finding alternative methods to introduce adhesion layers will be important for the practicality of O‐CSS and VTD methods.

Vapor transport deposition has also been used for Te_x_Se_1‐x_ [163, 164]. Lu et al. developed an approach using a transition of *a*‐Se rings to γ‐Te_x_Se_1‐x_ at 550°C [163]. While the temperature is too high for CMOS BEOL or flexible substrate integration, this result does give insights into crystal growth mechanisms within the system. In a follow‐up work, the same author then used polystyrene microsphere‐based lithography to create an inverse‐opal structured SnO_2_ ETL for light trapping purposes, then used the VTD they developed with Se and Te powder to form a Te_0.4_Se_0.6_ film [164]. Notably, the inverse‐opal substrate resulted in larger grain sizes and increased (101) texture compared to a planar compact SnO_2_ layer, in addition to the improved light management.

### 4.2 Solution Phase

Solution phase processing of chalcogenides is generally complicated by the lack of stable and readily available organoselenium and organotellurium compounds, particularly ones that would be suitable for preparation of elemental Se and Te. Further, the chemical stability of these elements limits the solubility in water or organic solvents. Generally, strongly reducing or oxidizing chemicals must be used to dissolve elemental chalcogenides.

#### 4.2.1 Hydrazine

Hydrazine (N_2_H_4_) is a strongly reducing solvent that is capable of dissolving elemental chalcogenides and metal chalcogenides via dimensional reduction [165, 166, 167]. In 2016, Zhu et al. fabricated solar cells by spin‐coating molecular precursors of elemental Se dissolved in hydrazine, using a c‐TiO_2_/mp‐TiO_2_ bilayer ETL and a PTAA HTL [68]. They showed that a chelating ethylenediamine (EDA) additive could control nucleation and improve morphology and device performance. Optimized devices achieved PCE as high as 3.52% under AM1.5G illumination. In a follow‐up work 2 years later, Zhu et al. used hydrazine to process Se films in ambient open air, making solar cells with up to 1.23% PCE [168]. Unfortunately, a comparison between the films processed in air and the films processed in a glove box was not provided. Nevertheless, hydrazine is an effective solvent for processing Se. However, it is certainly worth noting that hydrazine is exceptionally hazardous. Hydrazine and its derivatives are commonly used as rocket fuel, and it is highly explosive, environmentally hazardous, carcinogenic, toxic, and otherwise worth avoiding for both academic research and industrial processes whenever possible.

#### 4.2.2 Thiol‐Amine

Mixed thiol‐amine solvent systems have emerged as a safer and more flexible alternative to hydrazine for the solution processing of chalcogenides. Webber et al. showed in 2014 that thiol‐amine mixed solvents could effectively dissolve elemental Se and Te at atmospheric pressure without heating [169]. This included mixed solvents of ethylenediamine (EDA) and a thiol (e.g., ethanethiol (ET), and 1,2 ethanedithiol (EDT), 2‐mercaptoethanol). Similarly, many metal chalcogenides, metal oxides, and elemental metals can be dissolved in thiol‐amine solvent systems for processing of metal chalcogenides [170].

Later, Turnley et al. showed that *n*‐alklyamine:ET could produce an alkylammonium poly‐selenide (AAPSe) species [171]. This AAPSe precursor could be easily isolated as a solid and dissolved in a polar aprotic of choice. They used butylammonium poly‐selenide solutions as an “alkahest” to dissolve a wide range of metals for processing of kesterite and chalcopyrite materials and showed that the poly‐selenide species existed in a linear form, similar to that of hydrazinium poly‐selenides [172]. Desmukh et al. further showed that these AAPSe precursors can be used to dissolve Te [173].

Studies on thiol‐amine processing have almost exclusively focused on metal chalcogenides, but insights into precursor chemistry can be extended to make Se and Se‐Te alloy films. In 2022, Deshmukh et al. processed Se_1‐x_Te_x_ films with tunable bandgaps from EDA:ET [174]. They found that isolation of the Se‐Te species from excess EDA:ET prior to dilution in a mixture of EDA and a cosolvent was necessary to make more homogeneous films, with monoethanolamine (MEA) proving to be a better cosolvent than *n,n*‐dimethylformamide (DMF) and dimethylsulfoxide (DMSO). Finally, they made preliminary PVs from 30% Te films with 1.1% PCE. This work established thiol‐amine processing as a method to make Se and Te_x_Se_1‐x_ alloy films for optoelectronics. Further, this work demonstrated the importance of solvent engineering in this system, particularly the need to eliminate volatile thiol species from the deposition solvent. However, camera photos of the films (in the Supporting Information of the paper) suggest a rough surface, suggesting that improving the morphology of thiol‐amine processed films is necessary.

**FIGURE 8:**
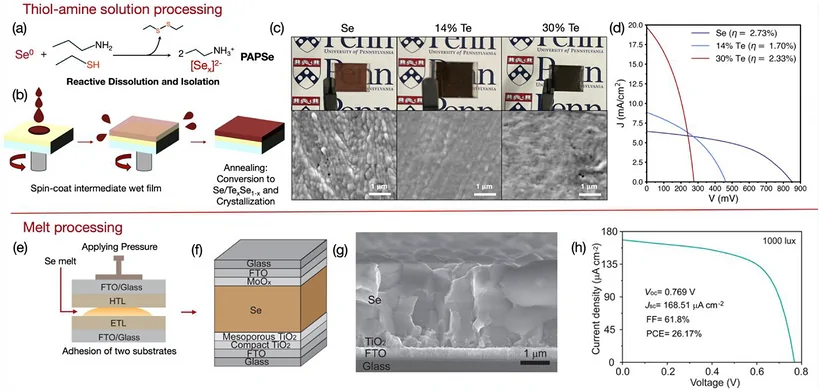
Solution and melt processing. (a) Schematic of the propylammonium polyselenide (PAPSe) preparation process: elemental Se is dissolved in propylamine and ethanethiol, then the resulting diethyldisulfide and unreacted PA/ET are removed to isolate solid PAPSe product. (b) Schematic method of depositing Se thin film from PAPSe ink. (c) Camera (top row) and SEM (bottom row) images of solution‐processed Se, Te_0.14_Se_0.86_, and Te_0.3_Se_0.7_ films. (d) J‐V curves of solution‐processed Se, Te_0.14_Se_0.86_, and Te_0.3_Se_0.7_ PVs under AM1.5G lighting. Schematics of (e) the melt lamination process and (f) the resulting bifacial devices. (g) Cross‐sectional SEM image of melt‐processed Se film. Films are significantly thicker than those processed by TE and solution processing. (h) J‐V curve of the melt‐processed Se PV under bifacial 1000 lux WLED illumination. These devices achieved a record efficiency for Se IPVs. (b‐d) Adapted under the terms of the CC‐BY Creative Commons Attribution 3.0 Unported license (https://creativecommons.org/licenses/by/3.0) [40]. (e‐h) Adapted with permission [41]. Copyright Wiley‐VCH GmbH.

Recently, we demonstrated that the AAPSe and poly‐selenotelluride species discovered by Turnley et al. make an excellent precursor for solution processing of Se and Se rich Se_1‐x_Te_x_ alloy thin films [40]. We spin‐coated inks consisting of propylammonium poly‐selenide (PAPSe, Figure 8a,b) and propylammonium poly‐selenotelluride (PAPST) in DMF with a MEA additive. PAPSe and PAPST precursors enabled a much wider processing window than films processed from EDA:ET, and we achieved uniform films (Figure 8c) with tunable bandgaps. We achieved PCEs of 2.73%, 1.70%, and 2.33% with pure Se, 14% Te, and 30% Te, respectively (Figure 8d), and sub‐200 nm thick Te_0.3_Se_0.7_ PDs showed 60% EQE in the visible range and ∼20% EQE at 900 nm. The PCE of the pure Se cells are comparable to those achieved with hydrazine, and the *V_oc_* > 850 mV is comparable to many vacuum‐processed films, but the PCE remains well below the 10% benchmark recently set by Wen et al., primarily due to a large *J_sc_* deficit. Solvent, additive, deposition, and interface engineering form a wide parameter space to control crystallization from AAPSe precursors, but significant improvements must be made to device performance before AAPSe‐processed PVs can be a viable alternative to vacuum processed cells.

Benny and Bhat took a similar approach, using MEA as the amine and ammonium thioglycolate (ATG) as the thiol to process Se and Se_1‐x_Te_x_ alloys for application in PVs [175]. However, due to the poor morphology, device efficiencies were below 0.1%. The benefit of using MEA:ATG or MEA:thioglycolic acid (TGA) routes for processing elemental chalcogenides is that these are greener chemicals that enable aqueous processing, but the film morphology from this precursor must be significantly improved. More insights into the various thiol‐amine precursors and their interactions with solvents (including water) are needed to develop this underexplored approach.

#### 4.2.3 Electrochemical

Se and Te can also be processed via aqueous electrochemical deposition (ECD). Solar cells consisting of TiO_2_ nanoparticles sensitized with an extremely thin (∼2 nm thick) ECD‐deposited Se layer achieved a remarkable 3.0% efficiency [176]. Here, the Se source was H_2_SeO_3_. Given the dominant role of the interface in these ultrathin absorbers and the sensitivity of PVs to interfacial defects, it would likely be necessary to further improve the interfacial passivation if this approach were to be revisited.

### 4.3 Melt Processing

Due to the low melting temperature of Se (∼221°C), melt‐based routes have also been explored for low‐cost processing [41, 177, 178]. The oxidative resistance of Se enables this process to be run in open air [177], and this melt processing is further compatible with fully printable devices [178]. The most successful application of melt processing was demonstrated by An et al., in which they used a hot‐pressing approach to laminate Se between two transport layer‐coated FTO/glass substrates to make bifacial Se PVs [41] (Figure 8e). In this method, one substrate was coated with a TiO_2_ ETL and the other with a MoO_x_ HTL (Figure 8f). The authors used an elevated melting temperature of 300°C to reduce the viscosity of the molten Se to achieve improved coating without excessive sublimation. Bifacial indoor PVs with dual side illumination (backside albedo of 0.8) reached >26% (Figure 8h), making this the most efficient Se IPV to date. The high efficiency approaches the desirable 30% efficiency mark, suggesting that Se is a serious candidate for IPV commercialization. It should be noted, however, that the films are quite thick (>2 µm, Figure 8g), consuming a significant amount of material. Further research on this melt lamination process should consider (i) reducing the thickness of the film to reduce material consumption and (ii) engineering the cooling and crystallization process.

## 5 Selenium Photovoltaics

### 5.1 Annealing Strategies for Se

As it stands, the 2‐step approach of TE and post‐annealing remains the most common approach to process Se due largely to its simplicity. The conventional approach for crystallizing amorphous TE‐deposited Se films is a 2–3 min anneal at approximately 200°°C on a hotplate in air. Metastable phases, high interfacial energies with commonly used transport layers, and a high vapor pressure make it challenging to achieve high crystallinity with the 2‐step process prior to sublimation or de‐wetting of the Se layer [69, 179]. A significant amount of the research in the Se PV field has therefore focused on understanding and engineering the annealing step.

**FIGURE 9:**
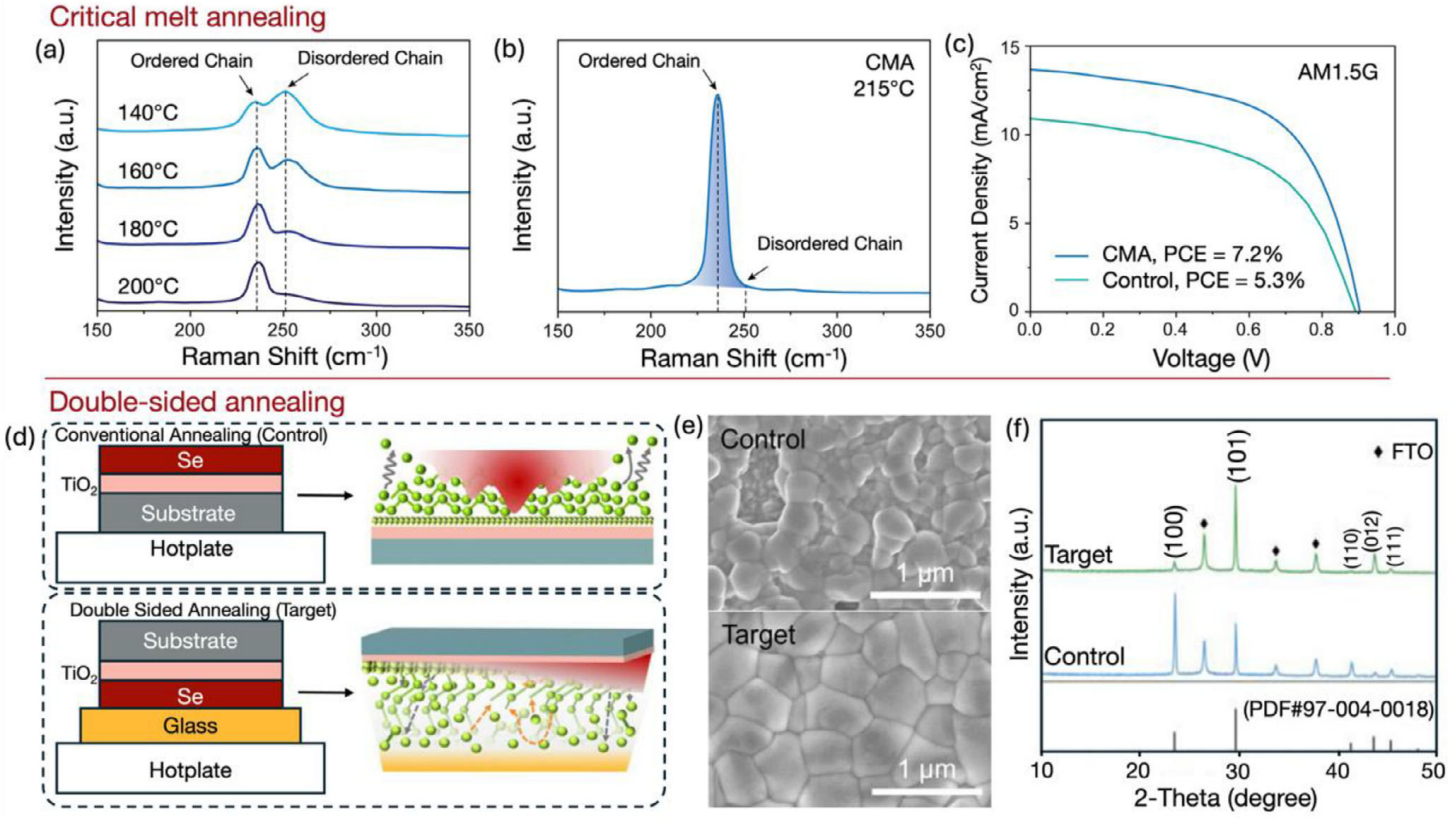
Annealing strategies. (a) Raman spectra of Se films at varying annealing temperatures showing a peak from ordered Se chains at a Raman shift of ∼235 cm^−1^ and disordered chains at a Raman shift of ∼251 cm^−1^. Annealing at temperatures 140°C to 200°C does not eliminate the disordered chain. (b) Raman spectrum of a Se film processed using the critical melt annealing (CMA) method (i.e., annealing at 215°C), eliminating the disordered chain signal. (c) Change in PV performance for Control (200°C annealing) devices and CMA‐processed devices. CMA shows a massive enhancement in *J_sc_* and performance. (d) Schematic of Control (top) vs. double‐sided annealing (DSA, bottom) process. (e) SEM images and (f) XRD spectra of Control (top) and Target (i.e., DSA, bottom) films. Grains are significantly larger and more uniform for DSA, and the films have better (101) texture. (a‐c) Adapted with permission [67]. Copyright 2024, Elsevier Inc. (d‐f) Adapted with permission [182]. Copyright 2026, Wiley‐VCH GmbH.

As discussed earlier, there is mounting evidence that disordered chains are a significant source of nonradiative recombination. Lu et al. used Raman spectroscopy to demonstrate that conventional annealing temperatures of 200°C do not sufficiently eliminate disordered chains (Figure 9a) [67]. They developed a “critical melt annealing” (CMA) technique to overcome this. They used a higher annealing temperature of 215°C, close to the melting point where the Se chains are more mobile. CMA eliminated the disordered chain signal in the Raman spectra (Figure 9b) and significantly improved the short circuit current density (*J_sc_*) relative to the conventional 200°C anneal. The PCE (AM1.5G) increased from 5.3% for the control to 7.2% for the CMA sample (Figure 9c). The CMA films enabled PCE_i_ of 18% under 1000 lux, and minimodules were constructed to power electronic shelf labels as a proof of concept. The PCE of unencapsulated devices remained constant for 1000h under AM1.5G illumination in ambient conditions with maximum power point tracking.

Duan et al. studied the effect of the annealing time (at 200°C) of evaporated Se on the film microstructure and PV performance [179]. Short (1 min) anneals at 200°C were insufficient to fully crystallize the Se film. Annealing at longer times (5 min) resulted in higher crystallinity and lower potential difference at the grain boundaries than films annealed for 2 min, but device defect densities were increased in the 5 min films relative to the 2 min annealed films. The authors attribute the increase in defects with time to Se sublimation. This study highlights the delicate balance between achieving high crystallinity and preventing Se vacancies.

Nielsen et al. addressed the tradeoff between crystallinity and Se loss by using a closed‐space annealing (CSA) technique: after crystallizing the Se with their standard 4 min 190°C on a hotplate and finishing the devices with MoO_x_ and Au, the device stacks are annealed at an elevated temperature of 210°C for 10 min [180]. The MoO_x_ in this process encapsulates the Se and prevents sublimation. This post‐anneal led to an increase in the short‐circuit current density, primarily by improving long‐wavelength photon collection. The proposed mechanism is improved contact between the MoO_x_ and Se, resulting in reduced parallel resistance. Surprisingly, despite the larger grain sizes and presumably improved crystallinity, there is no meaningful change in the carrier mobility or lifetimes. Another potential mechanism that was not explored in this work but could happen concurrently is a change in the interfacial chemistry at the Se/MoO_x_ interface [181].

Xia et al. similarly developed a modified annealing process designed to control the vapor pressure of Se during annealing for recrystallization of (101) textured films [182]. They used a double‐sided annealing (DSA) technique in which the Se substrate is placed Se film‐side down on a glass substrate on a hotplate during annealing (Figure 9d). A high temperature of 220°C results in a high vapor pressure of Se in this closed space, resulting in (101) recrystallization. This method resulted in larger grain sizes (Figure 9e), better texture (Figure 9f), and enhancement of the PCE from 4.86% to 6.50%. This approach demonstrates an innovative strategy to achieve (101) texture without modifying the vapor deposition process itself.

**FIGURE 10:**
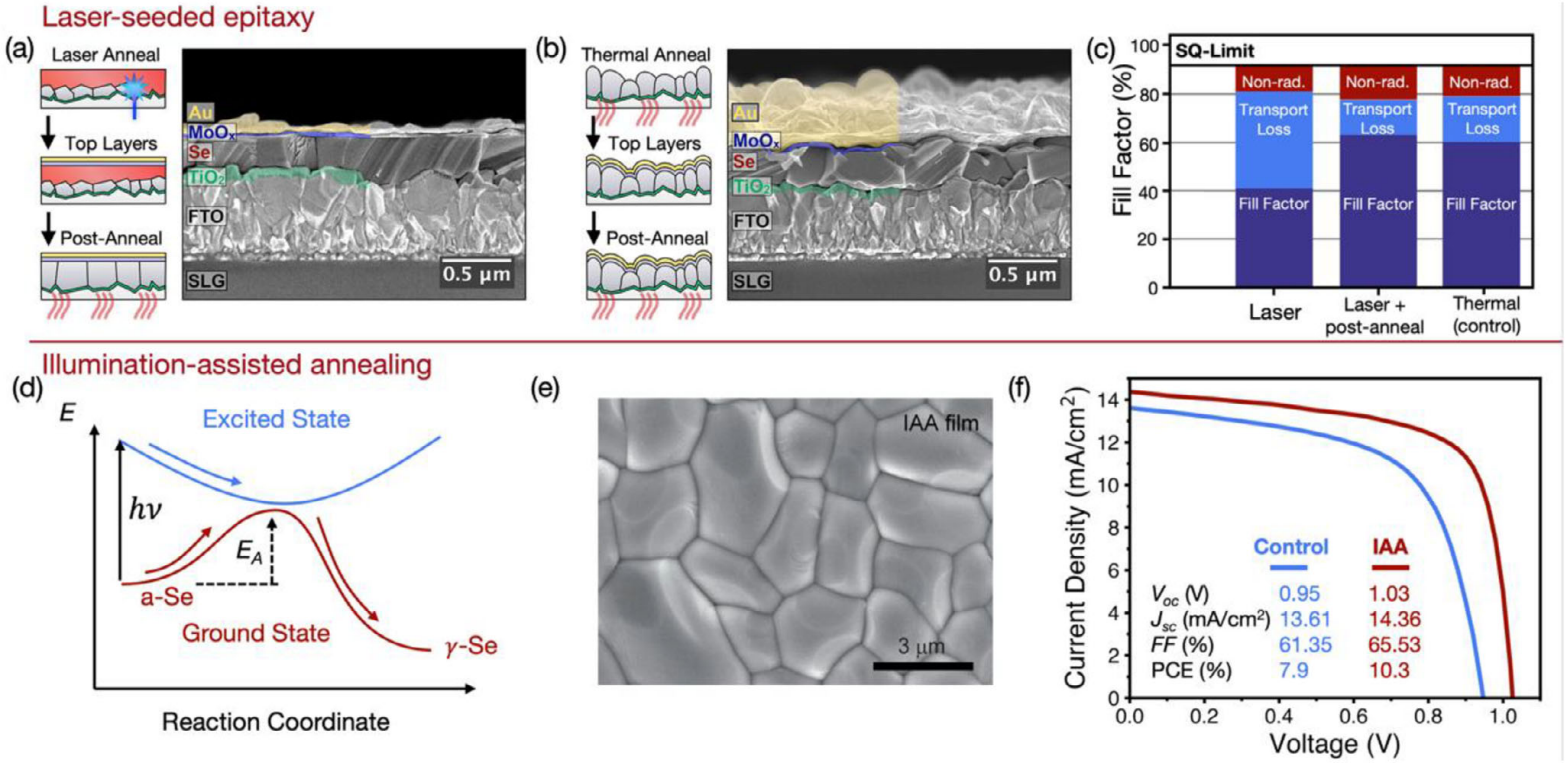
Laser Annealing and Illumination assisted annealing (IAA). Schematics and cross‐sectional SEM images of (a) laser annealing and (b) thermal annealing processes and films. (c) Origins of fill factor (FF) losses for devices from laser, laser + post anneal films, and thermally annealed films. (d) Schematic of how IAA assists in providing activation energy for crystallization. (e) SEM image of an IAA film, with significantly larger grains compared to conventional films and with no dewetting. (f) J‐V curves under AM1.5G lighting for control and IAA films. IAA films exceed 1V open circuit voltage (*V_oc_*) and 10% power conversion efficiency (PCE) for the first time in Se PVs. (a‐c) adapted with changes in fonts/colors with permission from ref. [34]. Copyright 2023, American Chemical Society. (d) is a recreation of a Figure in ref. [69]. (e) is an SEM image kindly provided by the authors of ref. [69]. (f) is a plot created using the data available in ref. [69]. For (d‐f) Copyright 2026, the Authors ref. [69].

An increasingly exciting approach to the materials processing of Se is the use of light as a degree of freedom. Hadar et al. presented results in 2019 that illuminating the Se with AM1.5G light during the crystallization anneal increases the Se film grain size and, accordingly, the PCE of the devices [152]. In 2023, Nielsen et al. proposed laser processing followed by solid phase epitaxy (SPE) as a method to enhance the quality of Se films [34]. In this work, they used a laser annealing process in which a 445 nm laser was rastered across the sample, incident through the ZnMgO/FTO/glass substrate. This creates a thin crystalline seed that serves as a template for SPE during a post‐annealing step (Figure 10a). This resulted in more uniform films with larger grain sizes and a better (101) texture compared to conventional thermally annealed films (Figure 10b). Resulting devices exhibited improved *FF* due to suppressed transport losses (Figure 10c).

Recently, Wen et al. demonstrated an illumination‐assisted annealing (IAA) approach that enabled the first demonstration of Se solar cells with PCE >10% and *V_oc_* > 1 V [69]. The key focus of this work was on increasing the grain size without inducing dewetting. The authors found that the high surface energy at the interface between Se and the ETL causes dewetting with extended annealing times. To mitigate this, they used a room‐temperature IAA approach for 24h under white light, before annealing for 2 min at 215°C. Here, the excited state produced by light absorption provides the activation energy needed for the transition from the metastable amorphous phase to the desired trigonal phase (Figure 10d). Illumination induces crystallization of the amorphous film, while the post‐anneal at 215°C drives grain growth, resulting in much larger grain sizes without delamination (Figure 10e). The reduced nonradiative recombination caused the *V_oc_* to improve from 0.95 V to 1.03 V, the *FF* to improve from 61.35% to 69.53%, and the PCE to improve from 7.9% to 10.3% (Figure 10f). All of these metrics are records for Se solar cells. This exciting result highlights the promise of Se as a PV material and underscores the value of illumination in enhancing the microstructure.

### 5.2 Interfaces and Device Engineering

Despite all the above improvements in annealing and film deposition, most of the device improvements come in the form of increased *J_sc_*, with only a handful of results changing the *V_oc_* by more than 50 mV. In fact, the largest *V_oc_* increases have been shown with improved device stack engineering. Todorov et al. increased the *V_oc_* by 158 mV by adding MoO_x_ as a HTL and another 83 mV by switching from the conventional TiO_2_ ETL to ZMO, which has a lower conduction band offset (CBO) with Se than TiO_2_ [183]. The n‐i‐p structure of ZMO/Se/MoO_x_ has become the highest‐performing architecture under AM1.5G illumination, and the ZMO/MoO_x_ combination is the only transport layer combination to reach *V_oc_* above 950 mV. TiO_2_ ETLs are still commonly used, particularly for IPVs. It is unclear if TiO_2_ exhibits an advantage over ZMO for IPV, however, as no results on Se IPV devices with ZMO have been published, to our knowledge. Table 2 lists device structures and their performances under solar simulation and, where applicable, indoor illumination.

**TABLE 2:** Transport layer engineering in Se PVs. This table is organized in descending order by PCE (AM1.5G), with the exception that studies that explicitly vary transport layers have the values grouped together. Only results with efficiencies above 5% are included, or results in which unconventional transport layers were used. All reported values for short‐circuit current density (*J_sc_*), fill factor (*FF*), and open‐circuit voltage (*V_oc_*) are for AM1.5G lighting.

Device Structure	Year	J_sc_ (mA/cm^2^)	FF (%)	V_oc_ (mV)	PCE (%) (AM1.5G)	PCE_i_ (%)	Ref
FTO/ZnMgO/Te/Se/MoO_x_/Au (n‐i‐p)	2026	14.36	69.53	1030	10.3	N/A	[69]
FTO/TiO_2_/Se/Au (n‐i‐p)	2024	14.37	60.61	930	8.10	N/A	[143]
FTO/TiO_2_/Te/Se/Au (n‐i‐p)	2025	12.58	64.16	936	7.55	17.36 (1000 lux 3000 K)	[29]
FTO/TiO_2_/Te/Se/Au (n‐i‐p)	2024	13.60	59.0	900	7.2	18.0 (1000 lux)	[67]
FTO/ZnMgO/Te/Se/MoOx/Au	2017	10.6	63.4	969	6.51	N/A	[183]
FTO/TiO2/Te/Se/MoOx/Au	2017	10.9	60.6	866	5.73	N/A	[183]
FTO/TiO_2_/Te/Se/Au	2017	10.5	50.5	728	3.88	N/A	[183]
FTO/ TiO_2_/Te/Se/Ti_3_C_2_T_x_	2026	14.26	54.72	833	6.50	12.2 (1000 lux)	[182]
FTO/ TiO_2_/Te/Se/Au	2026	11.74	52.96	781	4.86	N/A	[182]
ITO/PAA‐SnO2/Se/Au	2023	10.77	62.1	870	5.82	N/A	[184]
FTO/TiO_2_/Te/Se/MoO_x_/Au	2025	10.66	61.59	823	5.40	N/A	[181]
FTO/TiO_2_/Te/Se/MoO_x_/Au	2025	11.6	54.9	840	5.3	>8 (varied conditions)	[185]
FTO/TiO_2_/Te/Se/V_2_O_x_/Au	2025	11.4	55.0	830	5.2	>10 (varied conditions)	[185]
FTO/TiO_2_/Te/Se/WO_x_/Au	2025	10.1	52.8	810	4.5	N/A	[185]
FTO/TiO_2_/Te/Se/Au	2025	10.7	48.7	790	4.1	∼8 (varied conditions)	[185]
FTO/ZnMgO/Te/Se/MoO_x_/Au	2022	10.0	52.4	991	5.2	N/A	[42]
ITO/SnO2/Te/Se/P5NH/Au	2021	8.99	56.0	858	4.3	N/A	[186]
ITO/PTAA/Se/PCBM/Ag (p‐i‐n)	2024	9.76	52.8	900	4.64	N/A	[66]
ITO/PEDOT:PSS/Se/PCBM/Ag (p‐i‐n)	2020	9.61	47.8	839	3.9	N/A	[187]
ITO/ZnO/Se/Au (n‐i‐p)	2022	9.90	50.93	639	3.22	N/A	[47]
FTO/CdS/Te/Se/Au	2021	7.83	30.8	472	1.14	N/A	[188]

The MoO_x_ HTL is well known from organic, colloidal quantum dot, and Si heterojunction optoelectronics literature to have a large electron affinity and a large density of midgap states slightly below the conduction band [189, 190, 191, 192]. It is known that the work function of the metal contact gets pinned to these gap states, forming an excellent hole contact [193]. Recently, there have been new works that indicate the mechanism of the MoO_x_ HTL enhancement for Se in particular. Bao et al. showed that depositing MoO_x_ on Se while heating the Se film and substrate to 100°C results in the formation of a MoSe_x_ layer at the interface, facilitating charge extraction [181]. Segura‐blanch et al. found that this occurred at room temperature for MoO_x_ and observed that this could be generalized to V_2_O_x_ [185]. While MoO_x_ HTL devices performed better under AM1.5G lighting, V_2_O_x_ HTL devices exhibited higher PCE_i_ [185]. Another benefit of the use of the MoO_x_ HTL is that it enables the use of lower cost metals, such as Cu, that would otherwise diffuse into the film without the MoO_x_ barrier [194].

SCAPS simulations have suggested the importance of keeping the CBO below 0.2 eV to avoid *V_oc_* losses [13]. However, the typically used ETL, TiO_2_, has a CBO of ∼0.5 eV with Se, and ZMO has an offset of 0.4 eV [183]. This suggests that improving the band alignment of the ETL with Se while maintaining low transport losses could be a relatively simple approach to significant improvements in the *V_oc_*. Due to the intrinsic p‐type doping of Se, the ETL forms the p‐n heterojunction that dominates charge separation. We expect that optimizing this interface for reduced CBO and low defect densities is of greater urgency than the HTL, particularly because of the promising performance of high electron affinity transition metal oxide HTLs.

### 5.3 Benchmarking Se PVs

**FIGURE 11:**
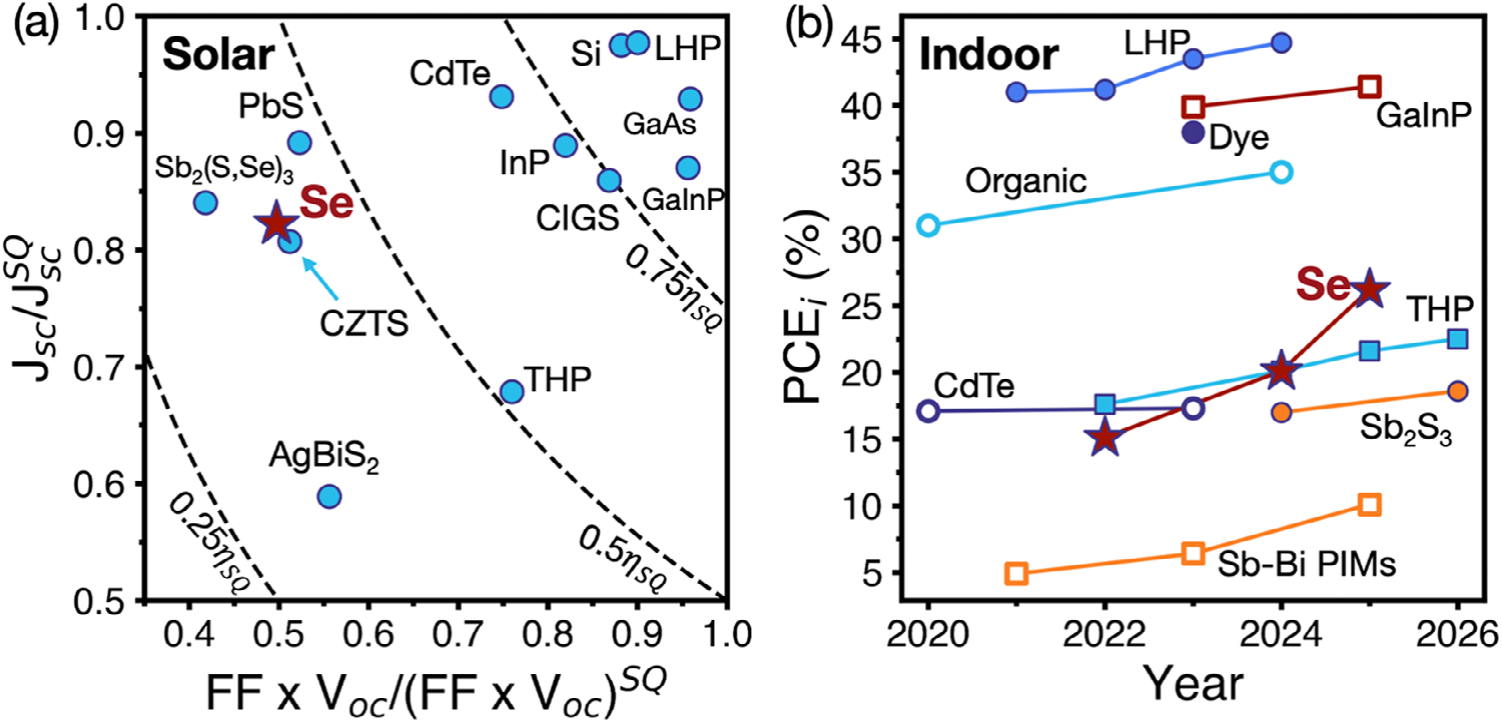
Benchmarking Se PVs. (a) J_sc_ and FF x V_oc_ as a fraction of the SQ limit for solar cells. LHP and THP denote lead halide perovskites and tin halide perovskites, respectively. CZTS and CIGS denote Cu_2_ZnSn(S, Se)_4_ and Cu(In, Ga)Se_2_, respectively. Sb‐Bi PIMs denotes mixed Sb and Bi perovskite inspired materials. Data for plot (a) taken from ref. [196] except for the following references, by material: Se [69], THP [197], AgBiS_2_ [198], PbS [199], and Sb_2_(S, Se)_3_ [200]. Data for plot (b) taken from the following references, by material: Se [14, 41, 149], LHP [87, 201, 202, 203], THP [88, 204, 205, 206], Sb_2_S_3_ [207, 208], Sb‐Bi PIMs [92, 209, 210], GaInP [211, 212], Dye [213], Organic [214, 215], CdTe [211, 216].

We compare the highest efficiency reported for Se PVs under AM1.5G lighting (from Wen et al. [69]) against efficiency records for other established and emerging inorganic or hybrid PV materials in terms of the *J_sc_* and the *FF* x *V_oc_* product compared to the Shockley‐Queisser (SQ) limits for each material (Figure 11a). *J_sc_*/*J_sc_* ^SQ^ is generally indicative of light management while (*FF* x *V_oc_*)/(*FF* x *V_oc_*)*^SQ^* is directly related to carrier management. Se sits below 50% of its SQ‐limited efficiency (η_*SQ*_), primarily due to *V_oc_* and *FF* deficits comparable to other emerging chalcogenide materials (AgBiS_2_, PbS, and Sb_2_(S,Se)_3_, and kesterites (i.e., CZTS)). Notably, Se has the largest bandgap of any material included on the plot, so it is well positioned for potential tandem solar cell applications.

In Figure 11b, we compare efficiency records for several IPV absorber materials by year since 2020. Se is ahead of tin halide perovskites (THPs), Sb_2_S_3_, and Sb‐Bi perovskite inspired materials (PIMs), making Se IPVs the most efficient RoHS compliant emerging inorganic thin‐film material candidate for IPVs. Se IPVs still trail LHPs, GaInP, organic, and dye‐sensitized IPVs. As previously discussed, LHPs have inherent toxicity challenges. GaInP is prohibitively expensive for IPV. Organic IPVs are promising, but the best‐performing devices are still processed with toxic halogenated solvents and expensive precursors [12]. Dye‐sensitized IPVs are a promising high‐efficiency, low‐cost, and non‐toxic option, but the highest‐performing dye cells still exhibit sustainability challenges related to commonly used redox couples and organic solvent electrolytes [12]. We argue that Se IPVs are the most promising inorganic option and are certainly worth continued development for low‐cost, high‐efficiency, and sustainable IPVs. Standardizing IPV testing and benchmarking performance is a current challenge in research due to the diversity of conditions under which IPVs operate and are tested [195]. We have included a table detailing the reported testing conditions for the IPVs in Figure 11b in the Table S1.

## 6 Te_x_Se_1‐x_ IR Photodiodes

### 6.1 Absorber Engineering

The typical composition for SWIR imaging is between 60%–70% Te, yielding a bandgap of approximately 0.7 eV. Although higher Te increases both the absorption coefficient in the SWIR and the carrier mobility, there is a tradeoff as hole concentration increases exponentially [20]. While this trend can generally be expected in semiconductors with decreasing bandgap, the typically observed hole concentration of ∼5 × 10^16^ cm^−3^ in Te_0.7_Se_0.3_ [18, 20] is 2–3 orders of magnitude higher than the expected intrinsic concentration of 10^13^–10^14^ cm^−3^ for a bandgap of 0.7 eV. This suggests that the increase in carrier concentration is due to charged shallow defects instead of thermally excited carriers. Further understanding of how composition influences defect and hole concentration is needed.

Typically, films are deposited using a similar 2‐step TE and crystallization anneal process as pure Se, though TE is done with pre‐alloyed Te_x_Se_1‐x_ and the annealing step is performed in an inert environment to prevent Te oxidation. In two studies that explicitly studied the effect of annealing temperature, monotonically increasing grain size and decreasing hole concentration are observed with temperature [18, 76]. However, higher temperatures above 260°C–280°C led to observations of film delamination, Se loss, and increased potential difference at grain boundaries. Consequently, mobility and device performance are found to be highest for films annealed in the range of 260°C–280°C. These observations suggest that mobility could be limited by either defects created by Se loss or by grown‐in strain introduced at higher annealing temperatures. We do note that R. Li et al. performed a similar study and achieved different results, identifying 200°C as the temperature at which carrier mobility and lifetime were the longest [20]. However, the annealing conditions in studies with the best‐performing devices are typically 250°C–260°C for 5 min [19, 22, 43].

Notably, the 260°C processing temperature is compatible with polyimide (PI) substrates. In addition to the low processing temperature, the mechanical properties of Te_0.7_Se_0.3_, stemming from the 1D vdW crystal structure, are suitable for flexible optoelectronics [96]. Pure Te has a large fracture strain of up to 30% along the [001] direction, and the vdW structure reduces the likelihood of dangling bonds forming upon fracture [96]. He et al. demonstrated flexible Te_0.7_Se_0.3_ PDs on PI with D^*^ = 4.8×10^10^ Jones and *R* = 0.29 A W^−1^ at 1300 nm, exhibiting minimal performance degradation after 1000 cycles of bending at a 5 mm bend radius [96], indicating the promise of Te_x_Se_1‐x_ PDs for wearable biomedical image sensors or other applications of flexible PDs.

### 6.2 Interfaces

Interfaces and transport layer engineering are important to enhance the efficiency, sensitivity, and speed of PDs while suppressing *J_d_*. In addition to introducing recombination sites, charge trapping at interfaces slows the response time and decreases the built‐in voltage (*V_bi_*), the latter of which degrades the rectification ratio and introduces higher *J_d_*. Most Te_x_Se_1‐x_ PDs use a ZnO or SnO_2_ ETL. There is less consensus at the back interface, where HTL‐free devices and devices with spiro‐OMeTAD and PTAA HTLs have been fabricated. Proper band alignment, defect passivation, and quick charge extraction are important for both interfaces.

**FIGURE 12:**
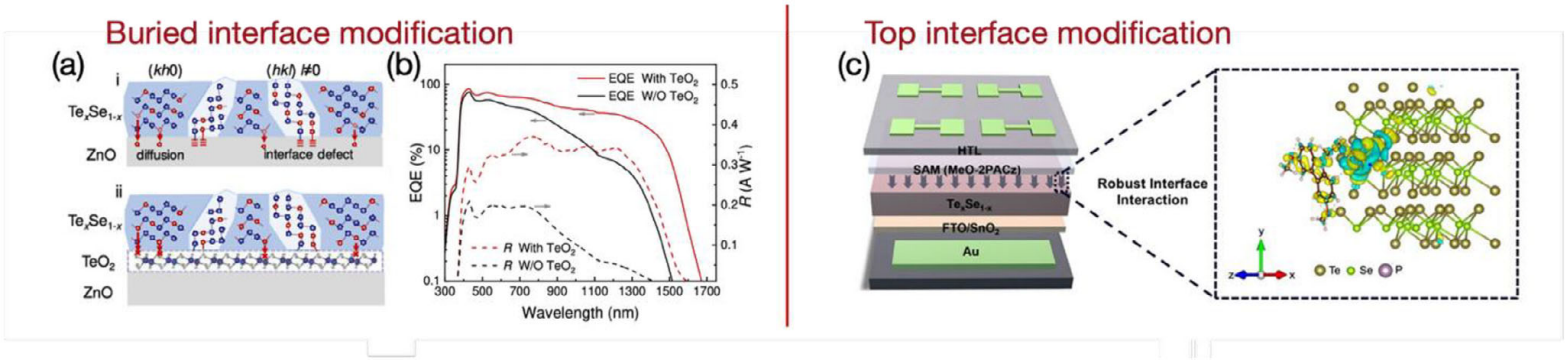
Advances in Interfaces for Te_x_Se_1‐x_ Photodiodes. (a) Schematic of how introducing a 0.3 nm thick TeO_2_ interlayer between ZnO and Te_x_Se_1‐x_ passivates interfacial defects from (101) oriented chains and prevents Se diffusion into ZnO. (b) EQE (solid lines) and *R* (dashed lines) spectra. (c) Schematic of Te_x_Se_1‐x_ PD device structure and (inset) the atomic scale interactions arising from introduction of the MeO‐2PACz SAM at the interface between Te_x_Se_1‐x_ and the PTAA HTL. (a‐b) Adapted with permission [19]. Copyright 2024, American Chemical Society. (c) Adapted with permission [22]. Copyright 2025, American Chemical Society.

Peng et al. demonstrated monolithic integration of a 64×64 pixel array of ZnO/Te_0.6_Se_0.4_ SWIR photodiodes with a Si ROIC [19]. A key demonstration in this work was the introduction of an ultrathin (0.3 nm) TeO_2_ interlayer between the ZnO and Te_0.6_Se_0.4_ to passivate dangling bonds at this interface and prevent Se and Te diffusion into the ZnO layer (Figure 12a). The addition of this interlayer decreased the interfacial defect density by an order of magnitude. Careful optimization of the TeO_2_ layer thickness significantly improved in the *R* at long wavelengths (Figure 12b), along with enhanced −3db bandwidth and D^*^.

Duan et al. took an alternative approach to modify the buried interface in devices where the n‐type material used was SnO_2_ [43]. They partially selenized the SnO_2_ prior to depositing the Te_0.73_Se_0.27_ absorber, improving the band alignment and passivating interfacial defects. The resulting devices had a larger built‐in voltage and suppressed interfacial trap density. Accordingly, the EQE was significantly enhanced relative to the control devices, and an improved LDR of 80 dB was achieved.

Hu et al. introduced a self‐assembled monolayer (SAM) of [2‐(3,6‐dimethoxy‐9H‐carbazol‐9‐yl)ethyl]phosphonic acid (MeO‐2PACz) between the Te_x_Se_1‐x_ active layer and the PTAA HTL [22] (Figure 12c). Strong interactions between the phosphonic acid group and the Se resulted in improved charge extraction and interfacial defect passivation. The HOMO of the SAM also was better matched to the VBM of the absorber, assisting in charge extraction and increasing the built in voltage. The addition of the SAM/HTL led to a marked increase in the EQE at 1320 nm from ∼9% to an impressive ∼20% (*R* of 0.49 A/W). Device response times <547 ns further solidify Te_x_Se_1‐x_ PDs as a high‐performance option for self‐powered photodetection. Together, the results from Duan et al. and Hu et al. highlight the importance of interfaces in enhancing the performance of Te_x_Se_1‐x_ PDs, meriting further studies related to interfacial layers and transport layer engineering.

### 6.3 Film‐Wafer Heterojunction Devices

Because Te_x_Se_1‐x_ can easily be integrated with a variety of substrates and many commonly used semiconductor wafers are transparent in the SWIR, PDs can be made using heterojunctions between Te_x_Se_1‐x_ thin films and semiconductor wafers. These device structures introduce new opportunities for monolithic integration, extending the *R* of Si photodetectors, and bias‐controlled spectral sensitivity by tuning band alignment [24].

Hu et al. demonstrated AZO/p‐ Te_0.75_Se_0.25_/n‐Si/AZO [23] PDs with bias‐tunable spectral sensitivity: negative bias enhanced the EQE of light absorbed in the Si close to the AZO/Si junction, while positive applied bias enhanced EQE of NIR/SWIR light absorbed in the Te_0.75_Se_0.25_ or in the Si close to the Si/ Te_0.75_Se_0.25_ interface. The Te_0.75_Se_0.25_ extended the spectral response compared to pure Si, enabling a D^*^ of 1.39×10^11^ Jones and a *R* of 0.063 A W^−1^ at 1550 nm. Notably, the −3dB bandwidth achieved was an impressive 280 kHz, and the response time was <1 µs. This work demonstrates how Te_x_Se_1‐x_ could be feasibly integrated with Si for optical communications and sensing. Te_x_Se_1‐x_/GaAs heterojunction PDs have also been demonstrated [217, 218]. However, the *R*, D^*^, and speed demonstrated in these devices have been similar or worse than all‐thin film Te_x_Se_1‐x_, and it is our opinion that the use of a III‐V wafer negates the primary cost and processing advantages of Te_x_Se_1‐x_. We believe that Te_x_Se_1‐x_ film‐wafer heterojunction SWIR PDs are most practical with Si wafers.

### 6.4 Benchmarking Te_x_Se_1‐x_ PDs

**FIGURE 13:**
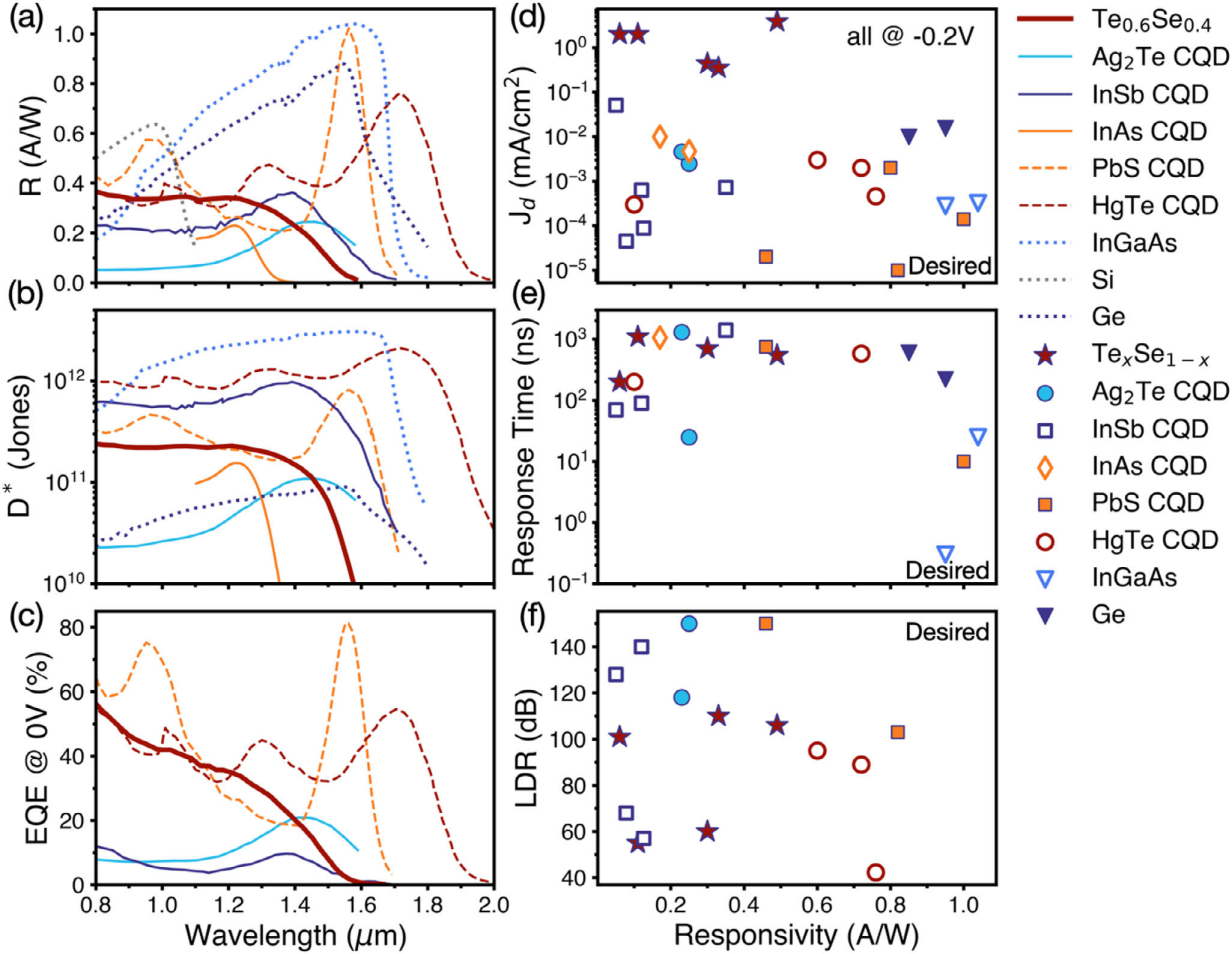
Benchmarking Te‐Se PDs. (a) Responsivity (*R*), (b) specific detectivity (D^*^), and (c) external quantum efficiency (EQE) at 0 V bias spectra for Te_0.6_Se_0.4_ [19], Ag_2_Te [112], InSb [219], InAs [221], PbS [121], HgTe [220] PDs from research and commercial InGaAs (model FGA21), Si (model FDS100), and Ge (model FDG03) PDs from ThorLabs [222]. CQD denotes colloidal quantum dot. D^*^ for Ge and InGaAs PDs is calculated using the listed active area and noise effective power at 1550 nm. EQE at 0V bias for the commercial PDs is not reported. (d‐f) show the dark current density (*J_d_*) at a bias of −0.2V (d), the response time (e), and the linear dynamic range (LDR) (f) and the *R* of various results for PDs from each technology. Data for (d–f) taken from ref. [18, 19, 22, 43, 76, 112, 121, 125, 126, 128, 219, 220, 223, 224, 225, 226, 227, 228, 229, 230].

Figure 13 compares the performance of Te_x_Se_1‐x_ SWIR PDs against commercial technologies (i.e., InGaAs and Ge PDs), mature CQD technologies in research (i.e., PbS and HgTe CQDs), and other emerging technologies (i.e., Ag_2_Te and In‐V CQDs). Figure 13a‐c show *R*, D^*^, and the EQE, respectively, as a function of wavelength for Te_0.6_Se_0.4_ [19] and other materials [112, 121, 219, 220]. *R* of Te_x_Se_1‐x_ SWIR PDs compares favorably to emerging CQD technologies, though the peak *R* for more established technologies is superior. D^*^ is superior to commercial Ge but remains slightly below other technologies. The EQE at 0V bias for Te_x_Se_1‐x_ is promising and is superior to that of emerging CQD PDs. Improving the EQE at longer wavelengths will be necessary, however. Figure 13d–f show the *J_d_* (at a small −0.2V reverse bias), response time, and LDR, respectively, plotted against *R* for various results for each material.

While the response time and LDR of Te_x_Se_1‐x_ PDs are comparable to other technologies, the *J_d_* is several orders of magnitude higher. As discussed in section 3.2.1, the high *J_d_* limits both *R* and D^*^ and is thus the greatest hurdle for Te_x_Se_1‐x_ PDs. Although the response time is comparable to or better than commercial Ge PDs and other technologies, the speed will need to be improved to be more competitive with the best InGaAs, PbS CQD, and Ag_2_Te CQD technologies. Finally, despite the noise introduced by the high *J_d_* and shunt conductance, the LDR is very promising, indicating that Te_x_Se_1‐x_ could be a highly sensitive technology for detection of weak signals with suppressed *J_d_*. Te_x_Se_1‐x_ PDs are surprisingly competitive with established technologies despite only 3 years of research. If *J_d_* can be reduced significantly, this technology has the possibility to be a high‐performance, low‐cost commercial technology.

## 7 Outlook, Challenges, and Conclusions

The fields of both Se PVs and Te_x_Se_1‐x_ SWIR PDs have seen major advancements in recent years. In the field of Se PVs, key advancements over the past 5 years include: (i) understanding of the Te interlayer, (ii) the importance and methods of achieving vertically oriented films, and (iii) better understanding and engineering of the annealing process. The *V_oc_* deficit remains the primary challenge.

Te_x_Se_1‐x_ SWIR photodiodes already offer promising performance that rivals commercial Ge PDs in D^*^ and response times after only 3 years of research, but high *J_d_* and low rectification ratios present a major challenge. In general, very little is known about the defects that influence performance in Te_x_Se_1‐x_ PDs, which inhibits calculated development of the technology for improved efficiency, sensitivity, and speed. Fundamental studies developing process‐structure‐property relationships in this material will be highly valuable to isolate the sources of nonradiative recombination, *J_d_*, and slower than desired response times.

### 7.1 Comparing Se PVs and Te_x_Se_1‐x_

We will briefly discuss similarities and differences between Se PVs and Te_x_Se_1‐x_ PDs. First, the entire Te‐Se system shares the same quasi‐1D crystal structure, resulting in anisotropic optoelectronic properties, though the extent of the electronic anisotropy is more severe for Se than Te‐rich alloys. Additionally, the existence of metastable ring‐like Se phases could impede the crystallization of amorphous Se films deposited by TE. Te‐rich phases do not contend with this challenge. Second, both systems exhibit signatures of extended defects of unclear origin. The addition of Te, however, increases the number of defect levels observed in electrical measurements, which could partly explain why Te‐Se alloys have poor *V_oc_* [35, 40]. Another difference in the photophysics that could impact carrier recombination is the stronger electron‐phonon interactions in Se relative to Te‐rich alloys.

There are certain advancements in process and device engineering that could be translated between fields. Notably, Te_x_Se_1‐x_ PDs have greatly benefited from engineering both the buried and top interface to passivate defects [19, 22, 43]. These strategies could potentially be of value to the Se PV community, where interfaces tend to have unfavorable surface energies [69, 152] and are possibly a significant source of nonradiative recombination. For Te_x_Se_1‐x_ PDs, annealing techniques developed for Se such as illumination assisted annealing, closed space annealing, and double‐sided annealing could be used to increase grain size, facilitate charge extraction rates, and control chalcogen volatilization. Moreover, the transition metal oxide hole contact layers that have been successful for Se PVs could be introduced in Te_x_Se_1‐x_ PDs.

The promise for both of these technologies relies on the prospects of low materials costs, low toxicity, and sustainability. We found that Se and Te indeed have low materials costs, but full technoeconomic analyses are needed to confirm that the low materials costs and scalable processing technologies used can transfer to manufacturing costs and, for Se absorbers in tandem solar cells, levelized cost of electricity. We similarly evaluated the toxicity and are in agreement with Nielsen that elemental Se and Te should be considered low toxicity materials rather than non‐toxic [27]. While there are no glaring toxicity or safety risks, a life cycle analysis for the technologies discussed would be necessary before eventual commercialization. Finally, we considered the fact that Se and Te are not abundant elements. Despite this, we conclude that Se IPVs and Te_x_Se_1‐x_ PDs can be scaled with little threat to global Se and Te reserves, and there is sufficient Se for several tens of TW capacity of hypothetical Se‐based tandem PVs before global supply becomes a limitation.

### 7.2 Scientific Challenges

#### 7.2.1 Shared Scientific Challenges

*Understanding defects*. Se and Te_x_Se_1‐x_ exhibit evidence of deep‐level states related to extended defects [44, 46, 65, 66], with the nature and processing origins of the defects remaining unclear. The impact of defects on device performance differs in these technologies. For Se PVs, the greater concern is non‐radiative recombination that causes a *V_oc_* deficit >490 mV relative to the radiative limit under solar irradiation, meaning elimination of deep trap states is of utmost importance. For Te_x_Se_1‐x_ PDs, the large *J_d_* is a greater concern, so the role of shallow defects on hole concentration and the role of defects in mediating dark current must be elucidated.

More studies combining advanced structural, theoretical, and device characterization techniques will be valuable. Developing a theoretical basis for extended defects in Se and Te_x_Se_1‐x_ and connecting this with advanced microscopy would help to fully understand the nature of defects in Se and Te_x_Se_1‐x_. Specifically, we suggest techniques that enable structural and optoelectronic information to be obtained simultaneously with high spatial resolution. This includes (i) TEM, SEM, or other electron beam microscopy techniques coupled with electron beam induced current and/or cathodoluminescence [231, 232, 233], and (ii) scanning probe techniques including C‐AFM, KPFM, and tip‐enhanced Raman and PL spectroscopy [234, 235]. It will then be valuable to connect these findings with optoelectronic characterization at the device level. This could include (i) temperature and power dependent *V_oc_* measurements to resolve contributions to *V_oc_* loss from interfaces, the space charge region, and the quasi‐neutral region [236], (ii) capacitance techniques that estimate defect densities (C‐V), energies (temperature dependent C‐f and DLTS), and depth (combined C‐V and DLCP) [65, 237], and (iii) transient photovoltage measurements that enable estimates of minority carrier lifetimes [67, 238]. For Te_x_Se_1‐x_ PDs in particular, dark J‐V measurements as a function of temperature would be valuable in identifying the origin of *J_d_* [239].

Modeling extended defects is far more challenging than understanding point defects, so this is nontrivial. We also believe that the role of point defects in Se should not be entirely disregarded. Point defects that do not influence lifetime can still impact diffusion lengths through impurity scattering. Additionally, extrinsic defects could potentially introduce mid‐gap states that could have large capture coefficients under certain doping conditions [64]. It is necessary to calculate capture cross sections for more extrinsic point defect types, specifically experimentally observed impurities such as pnictides and halides [44].

#### 7.2.2 Scientific Challenges for Se PVs

*Understanding Photophysics of Se*. It has long been known that carrier phonon coupling impacts carrier mobility in Se [48], but recent results indicate that carrier‐phonon coupling is directly influenced by variation in materials processing, with decreased electron‐phonon coupling correlated with improved *V_oc_* [46]. Because extended defects can introduce microscale strain and modify the phononic properties of materials, it is important to understand the interplay between carriers, phonons, and defects in Se.

In this context, the ultrafast photophysics of Se must be better understood. The ps decays in TAS photobleaching and OPTP photoconductivity could be explained by several phenomena, including trapping at extended defects, intrinsic carrier self‐trapping due to carrier‐phonon interactions [240], relaxation of carriers from the direct transition to the edges of the indirect bandgap, etc. The 1D structure, soft lattice, and large effects of acoustic phonon scattering on hole mobility increase the possibility that intrinsic trapping occurs [240]. However, unlike other materials that are known to have strong self‐trapped states [241], Se has minimal Stokes’ shift between the PL and absorption energies, and band edge TRPL has much longer lifetimes. More research is necessary into the photophysical processes in Se and how they relate to defects, carrier‐phonon interactions, and phonon‐defect interactions. This requires carefully designed ultrafast microscopy experiments that account for confounding variables and mimic real operational conditions.

#### 7.2.3 Scientific Challenges for Te_x_Se_1‐x_ PDs

The origin of shallow defects must be identified. Specifically, the extent to which *J_d_* can be suppressed depends on whether the high acceptor concentration results from (i) a minimal intrinsic defect concentration demanded by thermodynamics, or (ii) current processing conditions that introduce extrinsic or excessive levels of intrinsic defects.

*Understanding effects of crystalline orientation*. Claims of the (100) being advantageous for transport are questionable. While the carrier mobility in Te‐rich Te_x_Se_1‐x_ is less anisotropic than in pure Se and many other vdW materials, the mobility is still the highest along the (001) direction, so it is counterintuitive that the vertical transport in a (100) oriented device would be better than transport along the chains. For studies that claim a higher mobility enabled by (100) direction, Hall measurements are used. We note that, like OPTP, Hall measurements are in‐plane measurements and therefore will be along the direction of bonding for (100) oriented films, while transport will be out‐of‐plane for actual device operation. Hall measurements are therefore not meaningful in describing carrier mobilities for actual device applications of 1D vdW materials for PDs, and space charge limited conductance (SCLC) mobility of single carrier devices would be a far better metric. The (100) orientation may have the benefit of vdW bonding at interfaces, which could result in lower interfacial defect densities. In the studies claiming that (100) is superior, it would have been desirable to do C‐V and DLCP measurements to determine differences in interfacial defect densities. The (100) orientation may also have benefits in terms of enhancing absorption due to the higher imaginary permittivity for light polarized along chains. Therefore, while (100) orientation has led to the best device performance, it is important to fully understand the mechanisms when considering all factors and confounding variables.

### 7.3 Engineering Challenges

#### 7.3.1 Shared Engineering Challenges

*Uniformity and Crystallinity at scale.* Achieving uniformity and crystallinity at scale is important for both PV and PD applications. The anisotropy of the crystal structure means that crystalline orientation will need to be maintained across large films to minimize device‐to‐device variation. Combining deposition techniques that can achieve this while maintaining high throughput should be a focus.

#### 7.3.2 Engineering Challenges/Opportunities for Se

*Strategic Elimination of Extended Defects.* Once extended defects are better understood, engineering the processing steps can become more targeted. Bulk extended defects will likely be best resolved by improved engineering of the crystallization process (see below for potential directions), though post‐crystallization treatments to passivate defects or induce recrystallization could similarly be beneficial. Passivation treatments have been shown to be crucial in CIGS [242, 243] and CdTe [57, 244] PVs and should be explored in Se after crystallization processes are further developed. If grain boundaries act as a recombination site, it adds to motivation for a (003)‐oriented film, as the quasi‐1D van der Waals structure of Se enables self‐passivated, benign grain boundaries when the chains are oriented vertically [150, 151]. This has been shown to be accomplished using substrate heating during deposition and vapor‐mediated recrystallization, and more concepts could be adopted from the field of Sb_2_(S,Se)_3_ PVs [245, 246, 247]. Interfacial defects at the buried interface would require passivation treatments prior to deposition.

*Further development of light‐based annealing techniques.* From the work of Wen et al. [69], using light in the annealing process is clearly beneficial. We urge that other groups attempt to reproduce and refine this work while combining it with techniques to improve (*hk*1) texture. The simplicity of conventional processing is convenient, but it limits the control that can be exerted over the crystallization process. Introducing photonic annealing or illumination‐assisted annealing adds a degree of freedom to this process that opens new opportunities to manipulate crystallization kinetics and pathways.

*Beyond 2‐step processes for Se.* The conventional 2‐step TE + thermal anneal process is not well suited to avoiding metastable rings and associated phases, which may influence the optoelectronic quality of the resulting film. We therefore emphasize the need for more research into alternative deposition techniques that form Se directly in the desired γ phase instead of requiring solid phase crystallization. This includes vapor and solution phase approaches.

Preliminary work on vapor phase deposition of Se films using CSS is very promising, and this route is attractive as a high throughput vapor phase technique that directly grows the γ phase with the strong out‐of‐plane texture needed for optimal transport. Further understanding and optimization of the effects of various deposition parameters on film microstructure are necessary. The role of oxygen in enhancing grain size, in particular, requires further clarification. Similarly, VTD of Se films as a method to directly nucleate γ‐Se films with strong (003) texture is promising, but improved morphology and greater mechanistic understanding of pressure and gas flow rates are necessary. Additionally, we expect a variety of other PVD methods (pulsed laser deposition, sputtering, etc.) to be potentially viable.

We view solution processing as a promising route to develop this system, as the chain‐like structure of poly‐selenide precursors and high atomic mobilities in the solution phase suggest that direct crystallization in the γ‐Se phase with large grain sizes is possible. While efficiencies currently trail vapor processed Se PVs due to a large *J_sc_* deficit, solution processing could offer greater control over the crystallization process than is possible with conventional vapor phase processing. Further, solution processing offers higher throughput and lower capital costs than vacuum processing. However, to improve the crystallization process directly from solution phase, it is crucial to understand the fundamental processes of nucleation and growth from poly‐selenide based precursors and the interactions of these precursors with the solvent. Reducing the interfacial defect density in solution processed films will also be critical. We expect that the benefits of increased control and lower costs in the solution phase approach could outweigh the simplicity of vapor phase approaches, and we believe that the gap in efficiency of PVs processed by these methods will decrease and eventually vanish.

*Engineering the ETL.* The ETL choices of ZnMgO and TiO_2_ both exhibit CBOs >400 meV with the Se absorber, potentially causing *V_oc_* losses [13, 183]. Identifying an ETL material that forms a more ideal heterojunction partner without introducing series resistance or parasitic absorption is an underexplored but potentially important direction. It is worth noting that the ideal ETL for Se solar cells may differ from the ideal ETL for Se IPVs. The transport layers in general should also be considered in the context of the long‐term goal being to transition Se solar cells to tandem applications. Considering (i) the conventional n‐p polarity of Si and CIGS cells, which would be the most likely candidates for low bandgap materials, and (ii) the poorer performance of Se PVs when illuminated through the HTL side [41, 248], this would mean flipping the device stack deposition order to have the ETL deposited on top of Se, requiring new ETL materials to be explored for monolithic tandem PVs with Se top cells.

*Reduction in film thicknesses.* We mention in section 3.3 that developing Se solar cells for tandem applications could run into challenges with material availability at the multi‐TW scale. It is worth considering in the longer term how to minimize materials consumption via reduction of the absorber layer thickness without compromising efficiency. The highest efficiency devices typically have Se film thicknesses in the range of 800–1000 nm, but Todorov et al. showed almost a decade ago that Se absorbers of only 100 nm in thickness could still deliver >6% PCE [183]. Like other low dimensional chalcogenides [249, 250, 251], the complex refractive index of Se is quite high, meaning it can effectively localize and trap light. By introducing nanophotonic structures that introduce multiple resonances and near‐field coupled modes [252], it could be feasible to drastically decrease the thickness of Se to tens of nm without compromising light absorption. Scaling to these thicknesses introduces its own challenges, however, as interfacial states become more detrimental to device performance [253]. Scaling the thickness must therefore come with improved understanding of how to passivate interface states.

#### 7.3.3 Engineering Challenges/Opportunities for Te_x_Se_1‐x_

*Suppressing dark current.* A clear challenge when comparing the device performance to other technologies is the high *J_d_* and low rectification ratio. The shot noise‐limited D^*^ is inversely proportional to the square root of *J_d_* at low illumination intensities, and *J_d_* influences *R*. High shunt conductance, *G_sh_*, observed for devices with poor rectification, also influences the thermal noise. Hence, large *J_d_* and *G_sh_* limit D^*^ and *R*. The high *J_d_* is most problematic under reverse bias conditions. While self‐powered devices are often desirable to minimize power consumption, reverse bias can improve *R*, as this extends the depletion region and increases the electric field to assist charge separation. Reducing carrier concentrations and shunting pathways and improving the *V_bi_* through interfacial engineering should be expected to assist in reducing *J_d_* and *G_sh_*. This requires further understanding of the nature of the shallow defects that contribute to the high hole concentration (p ∼10^16^‐10^17^ cm^−3^) and the impacts of interfacial chemistry and band alignment on *V_bi_*.

*Enhancing response times*. While response times <550 ns have been achieved, the response times in Te_x_Se_1‐x_ PDs remain approximately an order of magnitude slower than the best CQD PDs and 1–3 orders of magnitude slower than commercial InGaAs PDs. Decreasing the device area (pixel size) will immediately enhance response times due to reduced capacitance, but work beyond this is necessary. By operating devices under reverse bias, the electric field in the absorber is increased and the junction capacitance is decreased, reducing the transit times and the RC delay. However, this requires suppression of *J_d_* to reduce idle power consumption and maintain high *R* and D^*^. In addition to reducing *J_d_*, decreasing the acceptor concentration will reduce the junction capacitance and RC delay. Trapping at interface states can also impede charge extraction, so continued work on optimizing interfaces and passivating interfacial defects will be needed to accelerate response times. Finally, decreasing the thickness of the absorber while leveraging light trapping techniques could be helpful to decrease transit times.

*Extending the range of responsivity.* The majority of Te_x_Se_1‐x_ PDs have been demonstrated with the best performance occurring at wavelengths of 1.32 µm and shorter. While this is a highly technologically relevant range, it will still be beneficial to extend further into the SWIR. The bandgap tunability of Te_x_Se_1‐x_ allows this, but achieving high *R* and low *J_d_* in lower bandgap materials will require further understanding of the origins of the high *J_d_* and how to suppress it.

### 7.4 Concluding Remarks

We are optimistic about the potential of Se absorbers for indoor and tandem photovoltaics and Te_x_Se_1‐x_ absorbers for SWIR photodiodes. However, both fields suffer from a poor understanding of the defects and photophysics that plague performance. For Se PVs, the open circuit voltage deficit is an obvious problem, while the key problem in Te_x_Se_1‐x_ PDs is the high dark current density. Identifying (i) the mechanisms responsible for these device level losses and (ii) their origins in materials processing steps or intrinsic properties is crucial for strategic development of these technologies.

## Author Contributions

**Adam D. Alfieri**: conceptualization, Writing – original draft, Writing – review and editing. **Deep Jariwala**: funding acquisition, writing – review and editing, supervision.

## Conflicts of Interest

The authors declare no conflicts of interest.

## Supporting information




**Supporting File**: advs77340‐sup‐0001‐SuppMat.docx.

## Data Availability

This work contains no original data to share.
