# Supplementary material for: Advances in Se Wide‐Bandgap Photovoltaics and TexSe1‐x SWIR Photodiodes
Source: Adv Sci (Weinh). 2026 Sep 3:e77340. Online ahead of print. doi: 10.1002/advs.77340 (PMC13540163; doi:10.1002/advs.77340)
Supplement: Supplementary file 1 [file ADVS-9999-e77340-s001.docx]

**Supplementary Information: Advances in Se Wide-Bandgap photovoltaics and Te_x_Se_1-x_ SWIR photodiodes**

Adam D. Alfieri^1,2^ and Deep Jariwala^1^*

^1^Electrical and Systems Engineering, University of Pennsylvania, Philadelphia, PA 19104

^2^CNRS-Institut Photovoltaïque d’Île de France (IPVF), UMR 9006, Palaiseau, France 91120

Table S1. Data for IPVs in Figure 11b. References refer to the references in the main text.

| **Material** | **Year** | **PCE_i_ (%)** | **Light Source** | **Intensity** | **Reference** |
| --- | --- | --- | --- | --- | --- |
| Se | 2022 | 15.1 | 2700K LED | 1000 lux | ^[149]^ |
| Se | 2024 | 20.1 | 6500K LED | 1000 lux | ^[14]^ |
| Se | 2025 | 26.2 | 6500K LED | 1000 lux, bifacial with 0.8 albedo | ^[41]^ |
| (CsMAFA)PbI_3-x_Br_x_ (LHP)* | 2021 | 41.0 | Fluorescent | 1000 lux | ^[201]^ |
| (CsFA)PbI_3-x_Br_x_ (LHP) | 2022 | 41.2 | Cool white LED | 1000 lux | ^[203]^ |
| (CsMAFA)PbI_3-x_Br_x_ (LHP) | 2023 | 43.5 | 3000K LED | 1000 lux | ^[87]^ |
| (CsMAFA)PbI_3-x_Br_x_ (LHP) | 2024 | 44.7 | U30 Fl | 1000 lux | ^[202]^ |
| GaInP | 2023 | 39.9 | 6000K LED | 500 lux | ^[211]^ |
| GaInP | 2025 | 41.4 | Cold white LED | 1000 lux | ^[212]^ |
| DSSC | 2023 | 38.0 | Fluorescent | 1000 lux | ^[213]^ |
| PM6:Y6-O (Organic) | 2020 | 31.0 | 3000K LED | 1650 lux | ^[214]^ |
| Organic tandem | 2024 | 35.0 | 2700K LED | 1000 lux | ^[215]^ |
| FASnI_2_Br (THP) | 2022 | 17.6 | 2956K LED | 1022 lux | ^[204]^ |
| FASnI_2_Br (THP) | 2024 | 20.1 | 3000K LED | 1000 lux | ^[206]^ |
| FASnI_2_Br (THP) | 2025 | 21.6 | 3000K LED | 1000 lux | ^[91]^ |
| FASnI_2_Br (THP) | 2026 | 22.5 | 3000K LED | 1000 lux | ^[88]^ |
| Sb_2_S_3_ | 2024 | 17.0 | 3000K LED | 1000 lux | ^[207]^ |
| Sb_2_S_3_ | 2026 | 18.6 | 3000K LED | 1000 lux | ^[208]^ |
| CdTe | 2020 | 17.1 | White LED | 1000 lux | ^[216]^ |
| CdTe | 2023 | 17.3 | 6000K LED | 500 lux | ^[211]^ |
| Cs_3_Sb_2_Cl_x_I_9-x_ (PIM) | 2021 | 4.9 | White LED | 1000 lux | ^[210]^ |
| (CsMAFA)_3_Sb_2_Cl_x_I_9-x_ (PIM) | 2023 | 6.4 | 4000K LED | 1000 lux | ^[209]^ |
| (CsMAFA)_3_(Sb,Bi)_2_Cl_x_I_9-x_ (PIM) | 2025 | 10.1 | 6500K LED | 1000 lux | ^[92]^ |

*FA = Formamidinium, MA = Methylammonium
